# Unlocking expanded flagellin perception through rational receptor engineering

**DOI:** 10.1038/s41477-025-02049-y

**Published:** 2025-07-28

**Authors:** Tianrun Li, Esteban Jarquin Bolaños, Danielle M. Stevens, Hanxu Sha, Daniil M. Prigozhin, Gitta Coaker

**Affiliations:** 1 Department of Plant Pathology, University of California Davis, Davis, CA USA; 2 Molecular Biophysics and Integrated Bioimaging Division, Lawrence Berkeley National Laboratory, Berkeley, CA USA; 3 Present Address: Plant and Microbial Biology, University of California Berkeley, Berkeley, CA USA

**Keywords:** Plant immunity, Plant biotechnology

## Abstract

The surface-localized receptor kinase FLS2 detects the flg22 epitope from bacterial flagella. FLS2 is conserved across land plants, but bacterial pathogens exhibit polymorphic flg22 epitopes. Most FLS2 homologues possess narrow perception ranges, but four with expanded perception have been identified. Using diversity analyses, AlphaFold modelling and amino acid properties, key residues enabling expanded recognition were mapped to FLS2’s concave surface, interacting with the co-receptor and polymorphic flg22 residues. Synthetic biology enabled engineering of expanded recognition from QvFLS2 (*Quercus variabilis*) into a homologue with canonical perception. A similar approach enabled transfer of *Agrobacterium* perception from FLS2^XL^ (*Vitis riparia*) into VrFLS2. Evolutionary analyses across three plant orders showed residues under positive selection aligning with those binding the co-receptor and flg22’s C terminus, suggesting more alleles with expanded perception exist. Our experimental data enabled the identification of specific receptor amino acid properties and AlphaFold3 metrics that facilitate predicting FLS2–flg22 recognition. This study provides a framework for rational receptor engineering to enhance pathogen restriction.

## Abstract (web summary)

Receptor kinase FLS2 detects the flg22 epitope of bacterial flagellin. Here the authors identify key residues on FLS2’s concave surface that enable expanded perception of flg22 variants, allowing the engineering of synthetic receptors with enhanced pathogen recognition.

## Main

Pattern recognition receptors (PRRs) on the plant cell surface can detect pathogens as non-self and mount a defence response. These receptors can recognize conserved microbial features known as microbe-associated molecular patterns (MAMPs)^1,2^. Leucine-rich repeat receptor kinases (LRR-RKs) are the most abundant and extensively studied type of surface receptor^2,3^. LRR-RKs have the capability to directly bind proteinaceous MAMPs^4^. Upon MAMP recognition, plant immune responses are initiated, including calcium influx, reactive oxygen species (ROS) production, phosphorylation of mitogen-activated protein kinase (MAPK) cascades, transcriptional reprogramming and callose deposition^5^.

One of the most well-studied bacterial-sensing PRRs is the LRR-RK Flagellin-sensing 2 (FLS2)^6^. FLS2 recognizes a conserved 22-amino acid immunogenic epitope (flg22) from the bacterial flagellin protein monomer^6,7^. Upon flg22 recognition, the SERK (Somatic Embryogenesis Receptor Kinase) family co-receptor BAK1–SERK3 binds to the C terminus of flg22, forming a complex with FLS2 (ref. ^8^). FLS2 is present across most land plants and has been characterized in diverse species, but most characterized homologues recognize epitopes similar to the canonical flg22 found in *Pseudomonas aeruginosa* (Pae)^9–12^. However, many α- and some β-proteobacteria possess non-immunogenic flg22 variants that escape immune recognition^13,14^.

Recently, FLS2 homologues with expanded ligand specificity have been discovered. For instance, FLS2 from a wild grapevine species *Vitis riparia* (FLS2^XL^)^15^, star jasmine *Trachelospermum jasminoides* (TjFLS2)^16^ and Chinese cork oak *Quercus variabilis* (QvFLS2)^16^ recognize polymorphic flg22 epitopes from *Agrobacterium tumefaciens*. Similarly, FLS2 homologues from *Glycine max* (GmFLS2s) specifically recognize flg22 from *Ralstonia solanacearum*, a devastating soil-borne bacterial pathogen^17^. Transgenic expression of FLS2^XL^ and the GmFLS2 receptor complex conferred resistance against *Agrobacterium* and *Ralstonia* pathogens in tobacco and tomato^15,17^, respectively, demonstrating the potential of leveraging expanded flg22 specificity for disease control.

In this study, we analysed the recognition profile of FLS2 homologues against polymorphic flg22 epitopes. Three novel FLS2 homologues (FLS2^XL^, TjFLS2 and QvFLS2) exhibit distinct recognition profiles and response magnitudes. Using a combination of sequence conservation analysis and AlphaFold3 modelling, we developed an approach to harness polymorphic residues on the receptor binding interface to engineer expanded ligand specificity. Evolutionary analysis and refined assessment of amino acid properties bolstered the engineering approach, demonstrating that certain FLS2 residues interacting with polymorphic flg22 residues and co-receptors are under positive selection. This indicates an evolutionary adaptation of FLS2 towards expanded ligand specificity and outlines a rational approach for receptor engineering.

## Results

### FLS2 homologues exhibit expanded flg22 ligand perception

**Fig. 1:**
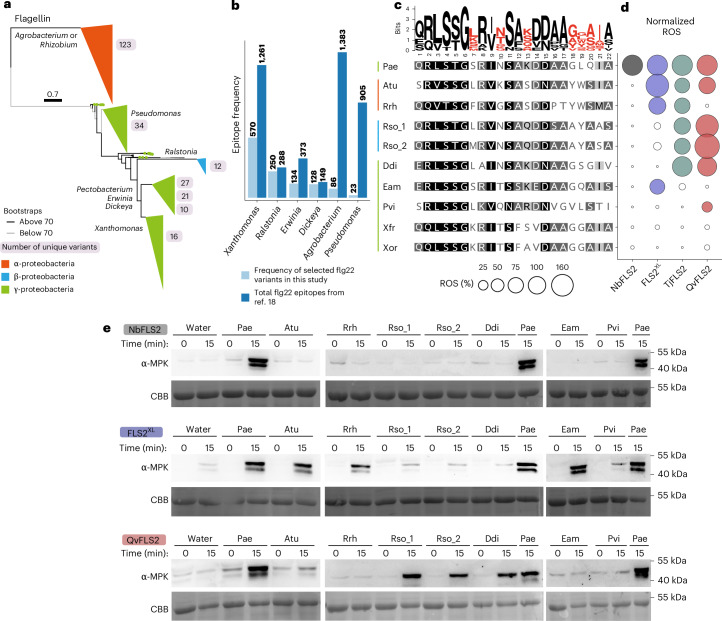
FLS2 homologues exhibit distinct perception range and magnitude. **a**, Maximum-likelihood, midpoint-rooted phylogenetic tree of bacterial flagellin. Major clades are collapsed in a genera-dependent manner and coloured on the basis of taxonomic classes. Branch thickness indicates bootstrap support. The number of unique flg22 peptide variants for each genus is given in a grey box. **b**, Frequency of flg22 variants characterized in this study in comparison with all flg22 variants mined in ref. ^18^. **c**, WebLogo and sequence alignment of flg22 from plant pathogenic bacteria, coloured bars represent different bacterial classes. **d**, Normalized ROS production after flg22 treatment (100 nM). NbFLS2 perception was analysed in wild-type *N. benthamiana*. Other FLS2 homologues were transiently expressed in the *N. benthamiana fls2-1/2* CRISPR–Cas9 mutant. Leaf disks for ROS assays were collected 24 h after infiltration. Normalized ROS levels were calculated by adjusting maximum relative light unit (RLU) averages to a 0 to 100 scale. Water was set to 0 and Pae flg22 was set to 100. Data points below a value of 10 are depicted as white bubbles. **e**, MAPK phosphorylation triggered by flg22 variants (100 nM). FLS2 expression occurred as described in **d** and tissue was collected 24 h after infiltration. Coomassie brilliant blue (CBB) staining indicates protein loading. All experiments were repeated at least three times independently with similar results. Nb, *Nicotiana benthamiana*; Qv, *Quercus variabilis*; Rso, *Ralstonia solanacearum*; Tj, *Trachelospermum jasminoides*; Xfr, *Xanthomonas fragariae*; Xor, *Xanthomonas oryzae*. Source data

**Extended Data Fig. 1:**
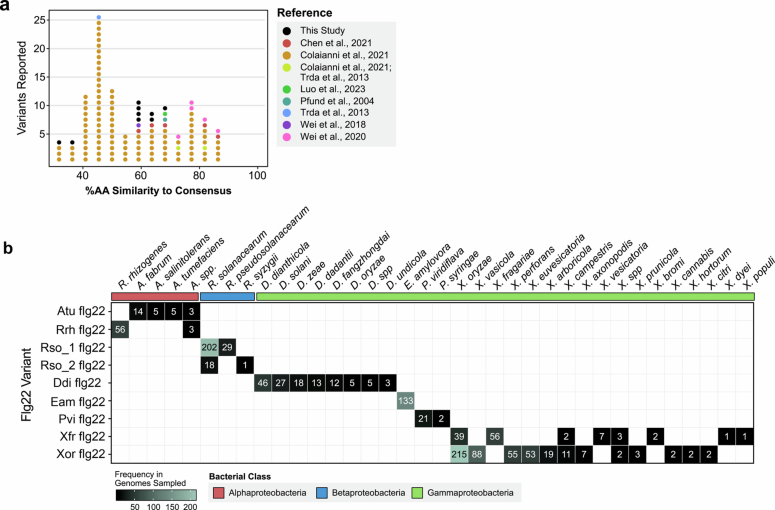
Diverse flg22 variants selected in this study. **a**, Percent amino acid (%AA) similarity of flg22 variants in comparison to the consensus selected in this study and past research. **b**, Frequency of selected flg22 variants tested in respect to their species designation from the genomes sampled.

To explore the extent of expanded ligand perception in different FLS2 homologues, we selected diverse flg22 epitopes from a comparative genomics study of 4,228 plant-associated bacterial genomes^18^. Ten polymorphic flg22 variants from important plant pathogens, representing α-, β- and γ-proteobacteria were chosen (Fig. 1a,b and Extended Data Fig. 1). These epitope variants exhibited 31.8% to 68.2% amino acid similarity to the consensus flg22 epitope from Pae and harboured C-terminal polymorphisms (Fig. 1c and Extended Data Fig. 1a). The selected flg22 variants exhibited diverse levels of abundance, but each was found in multiple bacterial species (Fig. 1b). Although most variants are present in multiple species, we used the most prevalent pathogenic species to represent each flg22 variant (Extended Data Fig. 1b).

**Extended Data Fig. 2:**
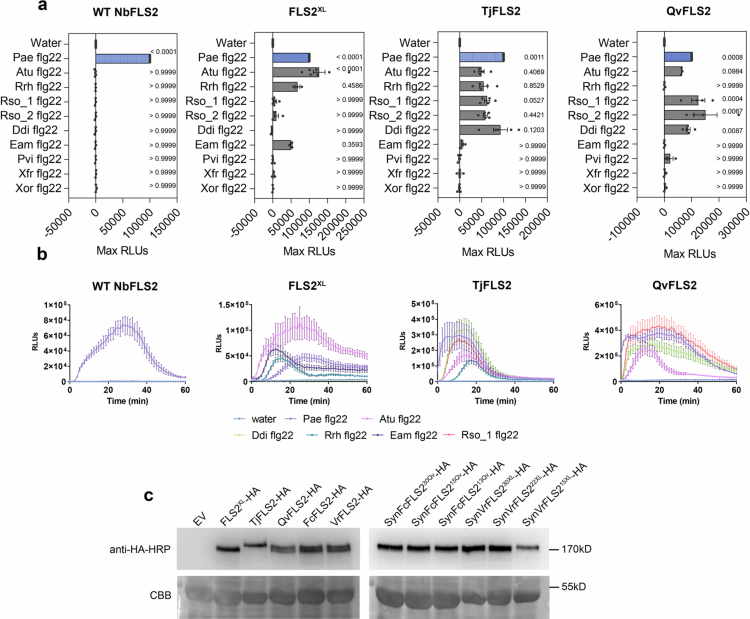
Perception of flg22 variants. **a**, Normalized ROS induction of flg22 variants (100 nM concentration) in WT *N. benthamiana* and the *N. benthamiana fls2-1/2* expressing *FLS2*^*XL*^, *TjFLS2* and *QvFLS2*. Each data point represents an average max relative light unit (RLU) from 4 plants with 4 leaf disks per plant. The values of each plate were normalized by adjusting maximum relative light unit (RLU) averages to a 0 (water) to 100000 (Pae) scale. One-way ANOVA and Dunnett’s multiple comparison were used to determine significance using the non-scaled data, *p* values are marked next to each column. Error bars = SEM. **b**, ROS curves for a subset of flg22 variants on various FLS2 expressing plants. Error bars = SEM, n = 4 leaf disks. All peptides were tested at 100 nM. **c**, Expression of FLS2 homologs and synthetic variants in this study. This experiment was conducted twice. CBB = protein loading. Source data

Next, the ability of FLS2 homologues from four species to perceive the 11 flg22 variants was characterized. Wild-type *Nicotiana benthamiana* was used to investigate perception of NbFLS2-1/2. The *N. benthamiana fls2-1/2* CRISPR–Cas9 mutant was used to investigate perception of FLS2^XL^, TjFLS2 and QvFLS2 using transient expression^16^. All FLS2 receptors could be detected by western blotting (Extended Data Fig. 2c). Using ROS as a proxy for recognition, we assayed perception profiles. FLS2 from wild-type *N. benthamiana* only recognized Pae flg22 (Fig. 1d and Extended Data Fig. 2a,b). By contrast, FLS2^XL^, TjFLS2 and QvFLS2 were able to recognize Pae flg22 in addition to other polymorphic variants (Fig. 1d and Extended Data Fig. 2a,b). FLS2^XL^ detects flg22 variants from three pathogens: *Agrobacterium tumefaciens* (Atu)*, Rhizobium rhizogenes* (Rrh) and *Erwinia amylovora* (Eam). TjFLS2 recognizes flg22 variants from Pae, Atu, Rrh, *Dickeya dianthicola* (Ddi) and two variants from *Ralstonia solanacearum*. QvFLS2 recognizes the variants from *Ralstonia solanacearum*, Ddi and *Pseudomonas viridiflava* (Pvi). No FLS2 homologues recognize flg22 variants from *Xanthomonas* species. Together, these results suggest that FLS2^XL^, TjFLS2 and QvFLS2 possess distinct expanded flg22 recognition profiles.

**Extended Data Fig. 3:**
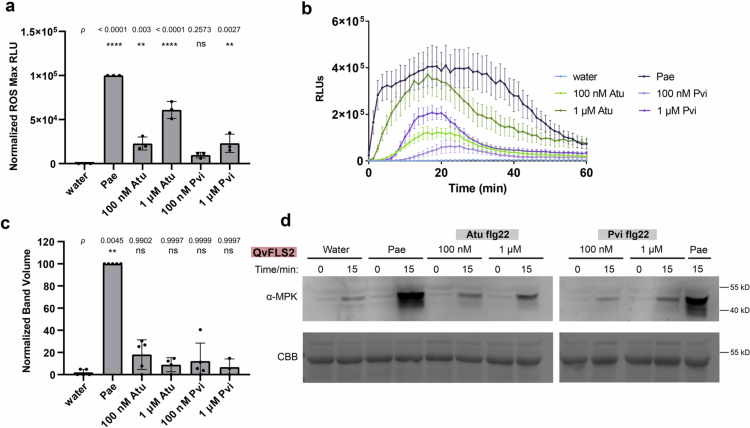
Deviant flg22s induce ROS but not MAPK phosphorylation. **a**, Normalized ROS induction of flg22 variants (100 nM concentration) in *N. benthamiana fls2-1/2* expressing QvFLS2. Each data point represents an average max relative light unit (RLU) from 4 plants with 4 leaf disks per plant. The values of each plate were normalized by adjusting maximum relative light unit (RLU) averages to a 0 (water) to 100000 (Pae) scale. A one-way ANOVA and Dunnett’s multiple comparison were used to determine significance using the non-scaled data, *p* values from left to right are marked on top. Error bars = SD. **b**, ROS curves for two different concentrations (100 nM and 1 μM) of Atu and Pvi. Data are presented as mean values ± SEM, n = 4 leaf disks. **c**, Normalized phosphorylated MAPK abundance. Bands from all treatments at 15 mins were normalized to Pae 15 mins on the same blot, then analyzed collectively. A one-way ANOVA and Dunnett’s multiple comparison were used to determine significance using the non-scaled data, *p* values from left to right are marked on top. Data are presented as mean values ± SD. Data points were derived from 10 independent blots, with each treatment repeated at least three times. **d**, Phosphorylation of MAPK induced by flg22 variants in the *N. benthamiana fls2-1/2* expressing QvFLS2. CBB = protein loading. All experiments were repeated at least three times with similar results. Source data

Perception of different FLS2 homologues was assessed using an additional immune output: MAPK phosphorylation (upon stimulation with 100 nM peptide). Generally, the outcomes from the MAPK assay aligned with those from the ROS assay (Fig. 1e). However, QvFLS2 induced a moderate ROS burst but no detectable MAPK phosphorylation in response to Atu and Pvi, even with a 10× higher peptide concentration (1 µM peptide) (Fig. 1d,e and Extended Data Fig. 3). This phenomenon has previously been described as ‘deviant peptides’, with an inconsistent immune output between the ROS assay and seedling growth inhibition^14^.

### Engineering of expanded flg22 perception from QvFLS2

**Fig. 2:**
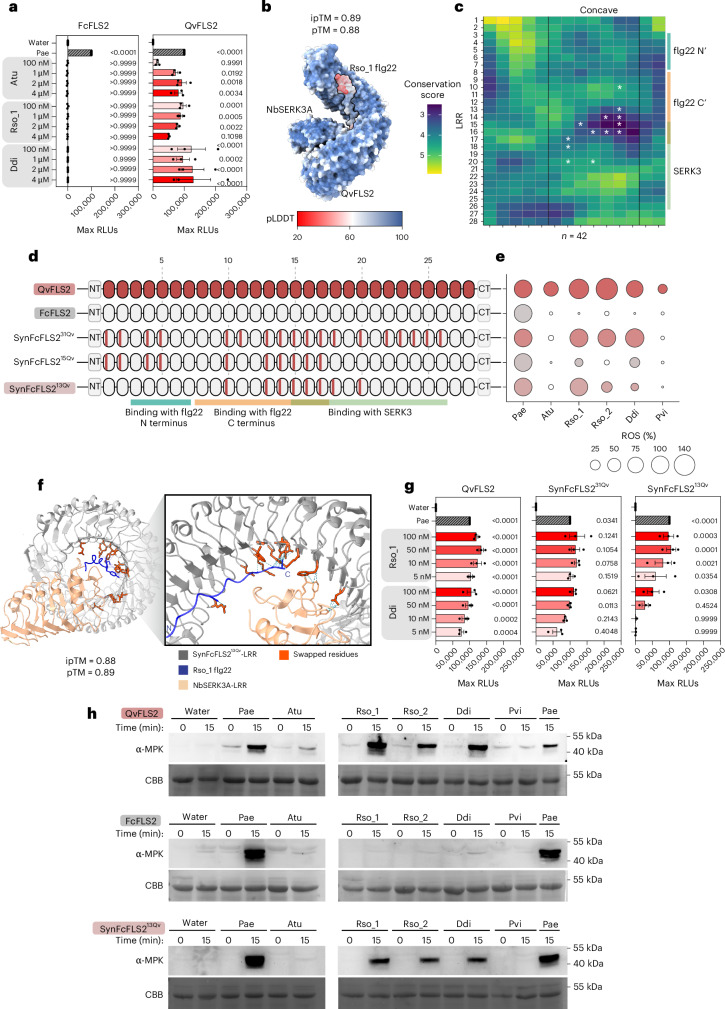
Engineering expanded ligand perception from QvFLS2 recognizing *Ralstonia* and *Dickeya* pathogens. **a**, ROS production after treatment with flg22 variants. FLS2s were transiently expressed in the *N. benthamiana fls2-1/2* CRISPR–Cas9 mutant. Each data point represents an average maximum RLU from four plants with four leaf disks per plant. Normalized ROS levels were calculated by adjusting maximum RLU averages to a 0 to 100,000 scale, referencing controls (water = 0 and Pae flg22 = 100,000) for each receptor. One-way analysis of variance and Dunnett’s multiple comparison were performed using non-scaled data, *P* values are marked for each column. Error bars denote s.e.m. **b**, AlphaFold3 model of the LRR extracellular domains of QvFLS2 and co-receptor NbSERK3A with *Ralstonia solanacearum* (Rso_1) flg22 peptide. The colour represents predicted local distance difference test (pLDDT). **c**, RCM of 42 FLS2 homologues in the Fagales order, showing conservation levels from high (yellow) to low (dark blue). White asterisks mark the residues transferred from QvFLS2 to FcFLS2 in synthetic FcFLS2 (SynFcFLS2^13Qv^), which are associated with the expanded ligand detection. Colour bars indicate interacting regions of the flg22 ligand or co-receptor SERK3. **d**, Representations of FLS2 variants. Colour bars indicate residue transfers. The numbers of residues transferred are superscripted. **e**, ROS production after treatment with flg22 variants (100 nM). FLS2s were transiently expressed in the *N. benthamiana fls2-1/2* CRISPR–Cas9 mutant. Normalized ROS levels were calculated by adjusting RLU averages to a 0 (water) to 100 (Pae flg22) scale. Data points below a value of 10 are depicted as white bubbles. **f**, AlphaFold3 model of SynFcFLS2^13Qv^ LRR and co-receptor NbSERK3A LRR with Rso_1 flg22 peptide. The N terminus and C terminus of flg22 are labelled. **g**, ROS production after treatment of flg22 variants with a concentration gradient as described in **a**. **h**, Phosphorylation of MAPK induced by flg22 variants (100 nM) for different receptors. All experiments were repeated independently at least three times with similar results. CBB staining indicates protein loading. C′, flg22 C terminus; CT, C-terminal LRR domain; Fc, *Fagus crenata*; N′, flg22 N terminus; NT, N-terminal LRR domain. Source data

We sought to engineer FLS2 with expanded flg22 recognition by introducing expanded specificity into homologues with narrow perception. QvFLS2 shares 66.80%–69.17% amino acid identity in its ectodomain with other homologues from diverse families (Fig. 1). We identified a homologue of QvFLS2 in the Fagaceae family, *Fagus crenata* (FcFLS2) as the engineering backbone. FcFLS2 shares 87.48% amino acid sequence identity with QvFLS2 but cannot perceive any tested polymorphic flg22 variants, even with a 40× higher peptide concentration (4 µM peptide) (Fig. 2a,e).

**Extended Data Fig. 4:**
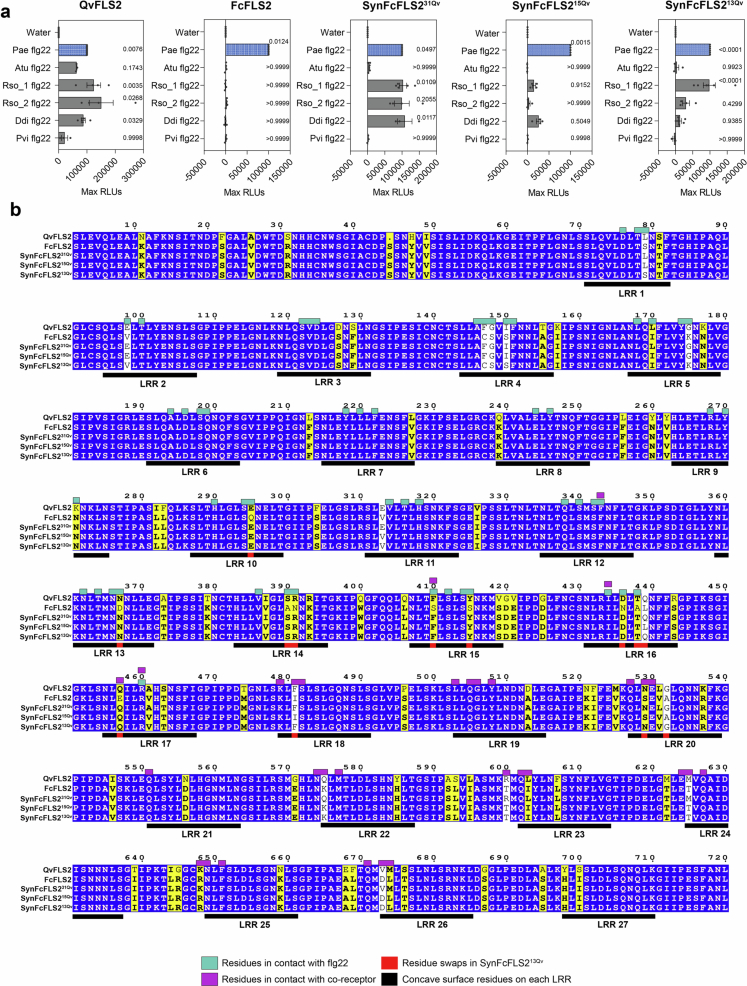
Engineering QvFLS2 specificity. **a**, Normalized ROS induction of flg22 variants (100 nM) in *N. benthamiana fls2-1/2* expressing QvFLS2, FcFLS2, and synthetic FcFLS2s. Each data point represents an average max relative light unit (RLU) from 4 plants with 4 leaf disks per plant. The values of each plate were normalized by adjusting maximum relative light unit (RLU) averages to a 0 (water) to 100000 (Pae) scale. One-way ANOVA and Dunnett’s multiple comparison were used to determine significance using the non-scaled data, *p* values are marked next to each bar. Error bars = SEM. **b**, Protein sequence alignment for LRR domains of five FLS2 variants. Concave surface regions of each LRR are highlighted with dark bars. Residues on the interacting surface are determined using the AlphaFold3 model of QvFLS2-NbSERK3A-Rso_1flg22 (< 5 Å). Residues in contact with flg22 are highlighted in cyan above the alignment, while residues in contact with the co-receptor SERK3 are highlighted in purple. Residues swapped for SynFcFLS2^13Qv^ are highlighted with red blocks.

The crystal structures of the LRR-RKs FLS2 and PEPR1 (PEP1 RECEPTOR 1) have highlighted the importance of concave-surface residues on the inner surface of the LRR superhelix for peptide ligand binding^8,19^. Therefore, we hypothesized that polymorphic residues on the concave surface contribute to receptor perception range. AlphaFold3 was used to model the complex of QvFLS2 with the co-receptor NbSERK3A and flg22 variant Rso_1 (Fig. 2b). We predicted the QvFLS2 residues required for interaction with Rso_1 flg22 as those within 5 Å (Extended Data Fig. 4b). We also used Repeat Conservation Mapping (RCM) to identify polymorphic residues in the LRR region^20^. QvFLS2 originates from *Q. variabilis*, a member of the Fagales order. Therefore, 42 FLS2 homologues from the Fagales order were subjected to RCM. A region with substantially lower conservation scores was identified between LRRs 12 and 20 on residues located on the concave surface (Fig. 2c). By contrast, LRRs 9–11 and 21–28 exhibited higher levels of conservation (Fig. 2c). Diverse FLS2 concave-surface residues identified by RCM are also predicted to interact with both the flg22 C terminus and co-receptor NbSERK3A. These data suggest that the expanded perception profile of QvFLS2 is primarily determined by variations in surface residues.

Next, we aimed to determine whether complete transfer of concave surface residues is sufficient to confer expanded ligand specificity. With 31 residue swaps from *Q. variabilis*, as indicated by the superscript number, SynFcFLS2^31Qv^ exhibited a similar ROS response magnitude against Rso_1, Rso_2 and Ddi flg22 as QvFLS2 (Fig. 2d,e and Extended Data Fig. 4a). However, it failed to recognize Atu and Pvi flg22, probably because of QvFLS2’s incomplete response to these variants (Fig. 2d,e and Extended Data Fig. 3). We then designed SynFcFLS2^15Qv^, which incorporates 15 residue swaps for QvFLS2 concave-surface residues that are predicted to primarily bind the entire Rso_1 flg22 region, omitting residues that solely bind to SERK3. All designs focused on residues that differ in hydrophobicity, charge and polarity. SynFcFLS2^15Qv^ encompassed residue swaps from LRRs 1 to 17 (Fig. 2d and Extended Data Fig. 4b). SynFcFLS2^15Qv^ exhibited a reduction in ROS response magnitude for Rso_1, Rso_2 and Ddi flg22, indicating diverse co-receptor binding residues are important for expanded recognition (Fig. 2d,e). Next, we generated SynFcFLS2^13Qv^, incorporating QvFLS2 concave-surface residues mainly in the flg22 C terminus and co-receptor binding regions (Fig. 2c,f). Using ROS as an output, SynFcFLS2^13Qv^ maintains a comparable response magnitude against Rso_1 flg22, but a reduced response to Rso_2 flg22 and Ddi flg22 (Fig. 2d,e Extended and Data Fig. 4a). Rso_2 flg22 and Ddi flg22 were still able to induce MAPK in plants expressing SynFcFLS2^13Qv^ (Fig. 2h).

To assess sensitivity, we performed ROS assays with different concentrations of flg22 epitopes from *Ralstonia* and *Dickeya* (100 nM to 5 nM) in Fig. 2g. SynFcFLS2^31Qv^ was able to perceive both epitopes at concentration of 5 nM but SynFcFLS2^13Qv^ was able to perceive Ddi epitopes at 50 nM (Fig. 2g). These data indicate that we were able to engineer expanded perception, but transfer of concave-surface residues did not result in enhanced sensitivity compared with the donor receptor. These data demonstrate rational receptor engineering of expanded ligand specificity by optimizing both the flg22-binding and SERK3-binding interfaces, guided by comparative analyses, protein modelling and amino acid properties.

### Engineering of *Agrobacterium* flg22 perception from FLS2^XL^

**Fig. 3:**
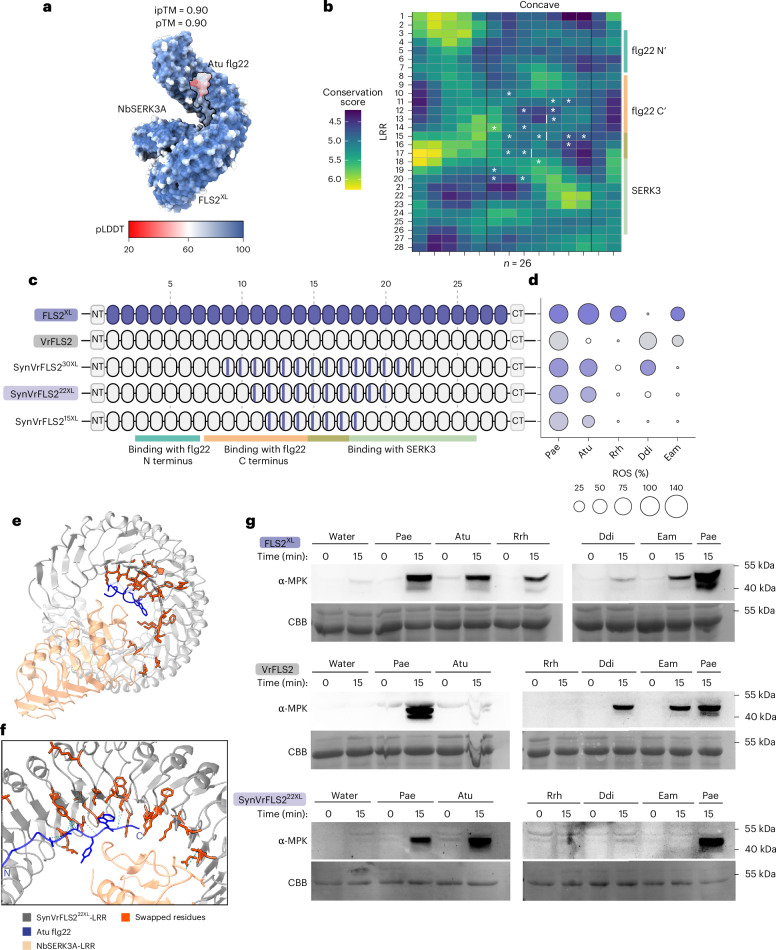
Engineering *Agrobacterium* flg22 perception by residue transfer from FLS2^XL^. **a**, AlphaFold3 model of the LRR extracellular domains of FLS2^XL^ and co-receptor NbSERK3A with Atu flg22 peptide. Colour represents pLDDT. **b**, RCM of 26 FLS2 homologues in the Vitales order, showing conservation levels from high (yellow) to low (dark blue). White asterisks and bars mark the residues transferred from FLS2^XL^ to VrFLS2 in synthetic VrFLS2 (SynFcFLS2^22XL^), which are associated with the altered ligand detection capability. Colour bars indicate the interacting regions of flg22 ligand or co-receptor SERK3. **c**, Representations of FLS2 variants. Colour bars in the LRRs indicate the occurrence of residue transfers. The numbers of residues transferred are superscripted. **d**, Normalized ROS production after treatment with flg22 variants (100 nM). FLS2s were transiently expressed in the *N. benthamiana fls2-1/2* CRISPR–Cas9 mutant. Normalized ROS levels were calculated by adjusting maximum RLU averages to a 0 (water) to 100 (Pae flg22) scale. Data points below a value of 10 are depicted as white bubbles. **e**,**f**, Top view (**e**) and binding interface (**f**) of AlphaFold3 model showing the LRR extracellular domain of SynFcFLS2^22XL^ and co-receptor NbSERK3A with Atu flg22 peptide. The N and C termini of flg22 are labelled. **g**, Phosphorylation of MAPK induced by flg22 variants (100 nM) for different receptors. CBB staining indicates protein loading. All experiments were repeated independently at least three times with similar results. Vr, *Vitis riparia*. Source data

**Extended Data Fig. 5:**
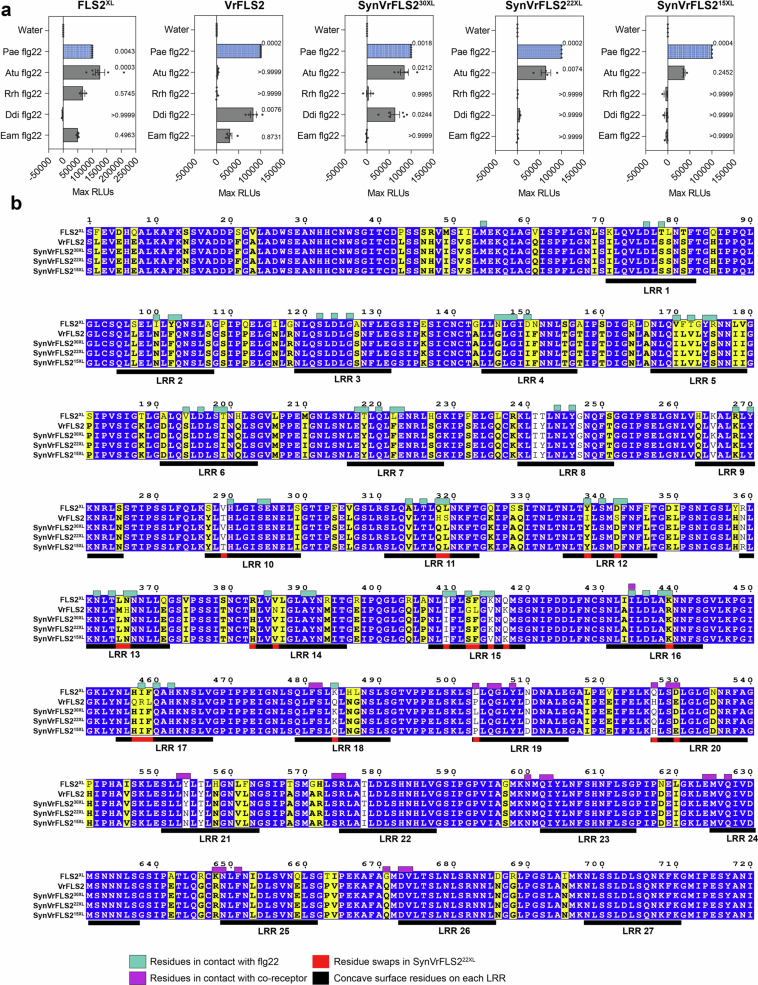
Engineering FLS2^XL^ specificity. **a**, Normalized ROS induction of flg22 variants (100 nM) in *N. benthamiana fls2-1/2* expressing FLS2^XL^, VrFLS2, and synthetic VrFLS2s. Each data point represents an average max relative light unit (RLU) from 4 plants with 4 leaf disks per plant. The values of each plate were normalized by adjusting maximum relative light unit (RLU) averages to a 0 (water) to 100000 (Pae) scale. One-way ANOVA and Dunnett’s multiple comparison were used to determine significance using the non-scaled data, *p* values are marked next to each bar. Error bars = SEM. **b**, Protein sequence alignment for LRR domains of five FLS2 variants. Concave surface regions of each LRR are highlighted with dark bars. Residues on the interacting surface are determined using the AlphaFold3 model of FLS2^XL^/NbSERK3A/Atuflg22 (< 5 Å). Residues in contact with flg22 are highlighted in cyan above the alignment, while residues in contact with the co-receptor SERK3 are highlighted in purple. Residues swapped for SynVrFLS2^22XL^ are highlighted with red blocks.

Next, we sought to engineer robust *Agrobacterium* flg22 specificity from FLS2^XL^ into its tandem repeat paralogue VrFLS2, which shares 82.07% protein sequence identity, using a similar approach. Like QvFLS2, diverse FLS2^XL^ concave-surface residues map to the flg22 C terminus and co-receptor binding regions based on AlphaFold3 modelling (Fig. 3a and Extended Data Fig. 5). VrFLS2 and FLS2^XL^ have distinct flg22 recognition profiles, except for shared Eam flg22 perception (Fig. 3c,d and Extended Data Fig. 5a). Owing to the limited diversity of sequenced genomes in the Vitales order, the diversity and conservation patterns on the RCM plot are less distinct than for QvFLS2^20^ (Figs. 2c and 3b). Nevertheless, we observed a similar region with lower conservation scores in LRRs 10–20, and highly conserved regions at LRRs 7–10 and 21–24. Complete LRR repeat swaps have demonstrated that FLS2^XL^ LRRs 12–18 are important for mediating Atu flg22 perception, aligning well with our RCM result^15^ (Fig. 3b).

To explore whether expanded recognition could be engineered, we generated three synthetic variants in the VrFLS2 backbone. Each SynVrFLS2 variant comprised residue swaps on the concave surface in regions predicted to interact with both the flg22 C terminus and co-receptor NbSERK3A (Fig. 3c and Extended Data Fig. 5b). Consistent with observations from QvFLS2 engineering, a gradual reduction in the number of residue swaps led to a decreased response magnitude of Atu flg22 (Fig. 3c,d and Extended Data Fig. 5a). Notably, by swapping 22 residues, SynVrFLS2^22XL^ effectively recognizes Atu flg22, triggering both a ROS burst and MAPK phosphorylation (Fig. 3). SynVrFLS2s gain Atu flg22 detection but lose Eam recognition, illustrating how structural changes enhancing one specificity can compromise another (Fig. 3c,d). These data also suggest that the residues underlying Eam perception are distinct in both receptors.

Collectively, these findings demonstrate that it is possible to engineer perception in two FLS2 receptors from diverse plant orders, each with distinct baseline recognition profiles. VrFLS2 begins with a weak, yet partially expanded recognition baseline, whereas FcFLS2 exhibits no expanded flg22 recognition (Figs. 2e and 3d). Using similar engineering approaches, we obtained different outcomes. SynFcFLS2 expanded its perception of three flg22 variants, whereas SynVrFLS2 gained Atu perception with a modified recognition profile (Figs. 2e and 3d). These results underscore the importance of testing the perception of multiple flg22 epitopes as well as considering the importance of each pathogen flg22 sequence for engineering recognition specificity.

### Surface residue properties correlate with ligand specificity

**Extended Data Fig. 6:**
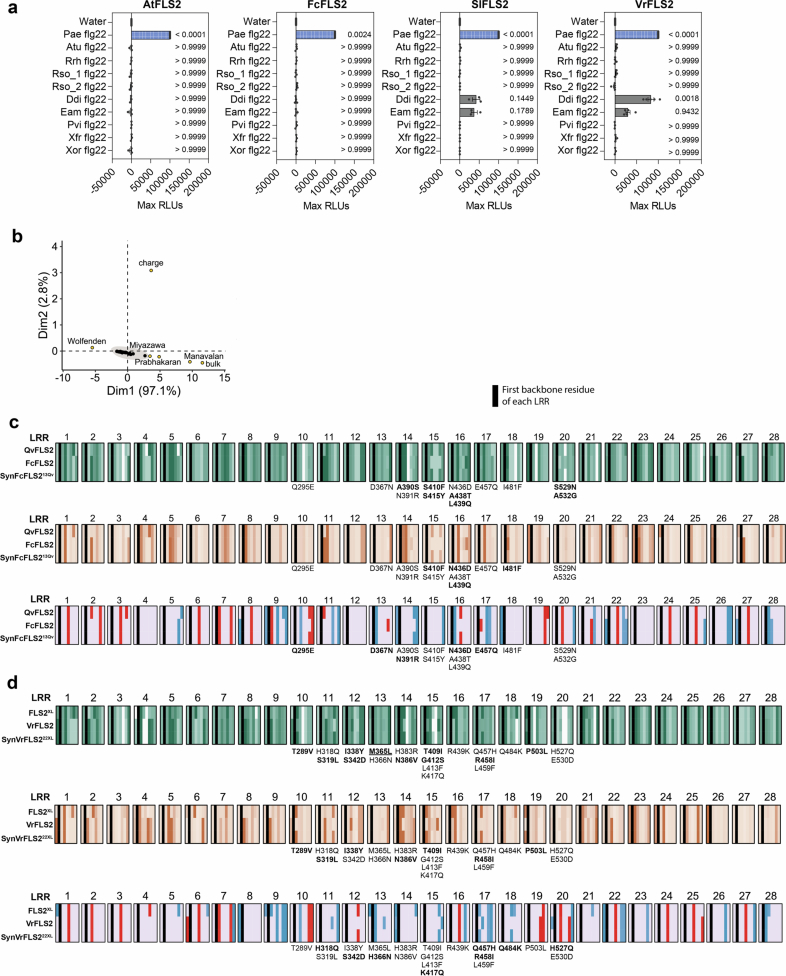
Principal component analyses identify amino acid chemical properties contributing to differential ligand perception. **a**, Normalized ROS induction of flg22 variants (100 nM) in *N. benthamiana fls2-1/2* expressing AtFLS2, SlFLS2, FcFLS2, VrFLS2. Each data point represents an average max relative light unit (RLU) from 4 plants with 4 leaf disks per plant. The values of each plate were normalized by adjusting maximum relative light unit (RLU) averages to a 0 (water) to 100000 (Pae) scale. One-way ANOVA and Dunnett’s multiple comparison were used to determine significance using the non-scaled data, *p* values are marked next to each bar. Error bars = SEM. At = *Arabidopsis thaliana*, Sl = *Solanum lycopersicum*, Fc = Fagus crenata, Vr = *Vitis riparia*. **b**, Principal component analysis (PCA) of FLS2 homologs and their associated residue properties. Scatterplot of contribution of chemical residue variables for top two PCA dimensions. Amino acid properties labeled in yellow display the highest variance and those in black display little variance between FLS2 homologs. **c-d**, Heatmap of bulkiness (top), hydrophobicity (middle), amino acid charge (bottom) along exposed residues of the LRR across FLS2 variants. All swapped residues in SynFcFLS2^13Qv^ (**c**) or SynVrFLS2^22XL^ (**d**) are labeled below the heatmaps. Swapped Residues with substantial change of chemical parameters are highlighted in bold (Δ bulkiness > ±2, Δ hydrophobicity > ±2, Δ charge > ± 0.5). The underlined residue is not directly located on the concave surface, yet was selected for engineering based on changes in side chain properties.

Diverse amino acid properties influence the binding potential of protein–peptide interactions^21,22^. We demonstrated that residues in the concave surface are critical for mediating perception of diverse flg22 peptides (Figs. 2 and 3). Next, we investigated how individual amino acid properties contribute to binding potential and perception range. We selected eight FLS2 homologues with canonical (Pae) or expanded perception of between two and five ligands (Fig. 1d and Extended Data Figs. 2a and 6). We found that *Solanum lycopersicum* (tomato cv Money Maker) SlFLS2 can also perceive Ddi and Eam flg22 and is categorized as expanded perception (Extended Data Fig. 6a). Using an unbiased approach, average chemical values of exposed residues along the concave surface were calculated. This analysis encompassed 44 distinct amino acid properties, including bulkiness, charge, aliphatic index and multiple hydrophobicity scales. Different hydrophobicity scales are based on experimental measurement, statistical analysis of their occurrence in proteins, and computational models predicting their interactions with water and other molecules^23^.

**Fig. 4:**
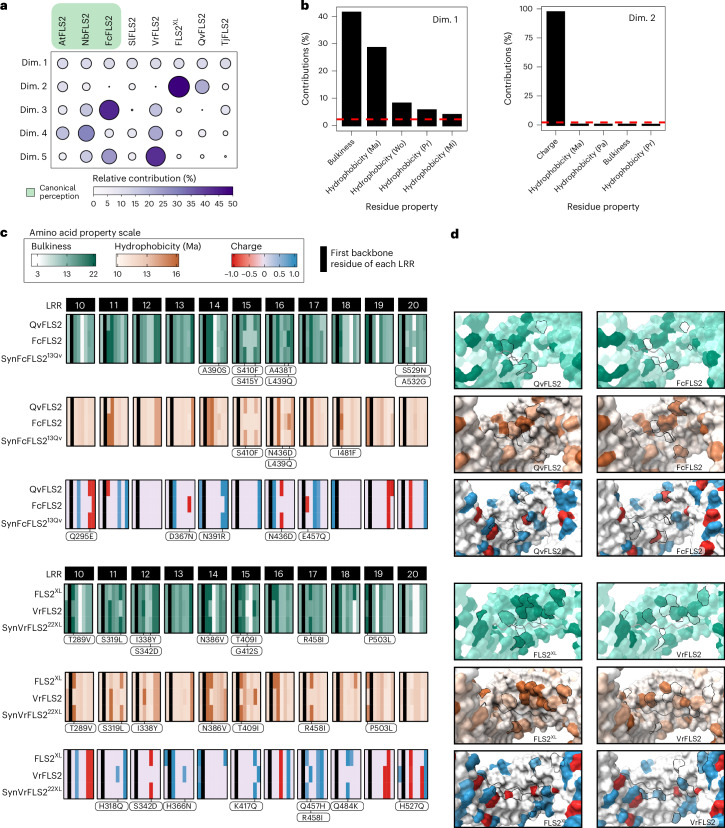
PCA identifies amino acid properties contributing to expanded ligand perception. **a**, PCA of FLS2 homologues and their associated residue properties. The first five dimensions are displayed (dimensions (Dim.) 1 to 5) with their relative contributions across each FLS2 homologue. **b**, Highest ranked contribution of chemical residue variables for top two PCA dimensions. **c**, Heatmap of bulkiness (top), hydrophobicity (middle) and amino acid charge (bottom) along exposed residues of the LRR for different FLS2 variants. Residue swaps in synthetic variants that display differential bulkiness and/or charge are denoted (Δ bulkiness more than ±2, Δ hydrophobicity more than ±2, Δ charge more than ±0.5), with dark lines connected to the corresponding residue in the heatmaps. Surface-exposed residues are defined as ‘xLxxLxLxxNx’ of each LRR. **d**, AlphaFold3 model of the LRR regions of FLS2^XL^, VrFLS2, QvFLS2 and FcFLS2 with residues coloured by bulkiness or charge as labelled in **c**. Swaps of interest are outlined in black. At, *Arabidopsis thaliana*, Sl, *Solanum lycopersicum*. Ma, Manavalan; Mi, Miyazawa; Pa, Parker; Pr, Prabhakaran; Wo, Wolfenden.

From the average values across all 44 properties, a principal component analysis (PCA) was performed to identify which amino acid properties displayed the greatest variation across receptors and correlated with receptor perception profiles (Extended Data Fig. 6a). Dimension one explained the greatest proportion of the variance (97.1%) across all FLS2 homologues, but the relative contribution per homologue was similar (Fig. 4a). Assessing the relative contribution of individual chemical parameters for dimension one revealed that bulkiness and hydrophobicity (using the Manavalan scale) predominately contributed (Fig. 4b). The Manavalan scale considers the hydrophobicity of nearby residues commonly found in globular proteins^24^. Dimension two accounted for 2.8% of the total variance, with charge being the primary contributing factor (Fig. 4b). Strikingly, the relative contribution of dimension two varied greatly across FLS2 homologues, with it being the strongest contributor to variance for FLS2^XL^ and QvFLS2 (Fig. 4a).

Next, we investigated differences in bulkiness, hydrophobicity and charge values of concave-surface-exposed residues between FLS2 homologues with canonical, expanded and engineered expanded perception (Fig. 4c,d and Extended Data Fig. 6b,c). We focused on LRRs 10–20, which bind flg22’s C terminus, the SERK co-receptor, and are sufficient for engineering expanded perception. Bulky and charged residues differ in location for QvFLS2 and its canonical counterpart, FcFLS2 (Fig. 4a,b). The residue profile of SynFcFLS2^13Qv^ closely matched that of QvFLS2 for bulkiness and hydrophobicity in LRRs 14–20, and for charge in LRRs 10–17 (Fig. 4c and Supplementary Table 1). Overall, FLS2^XL^ carried more bulky and hydrophobic residues than its canonical counterpart VrFLS2 between LRRs 10 and 19 (Fig. 4c). The bulky residue profile of SynVrFLS2^22XL^ closely matched that of FLS2^XL^ (Fig. 4c and Supplementary Table 1). Alterations in charge between FLS2^XL^ and VrFLS2 generally occurred in LRRs 11–18 (Fig. 4c). The charge profile of SynVrFLS2^22XL^ closely matched that of FLS2^XL^ (Fig. 4c). Broadly, pairing amino acid properties with protein modelling will enable more-precise modifications to adjust epitope recognition profiles.

### Predicting flg22 recognition using AlphaFold3

Using confidence scores from AlphaFold has facilitated the identification of protein binding interfaces^25^. Although most research has focused on structured proteins, the efficacy of AlphaFold in recognizing peptide ligands remains less explored. We sought to determine whether AlphaFold3 is capable of accurately predicting recognition of diverse flg22 variants across different FLS2 receptors. If successful, AlphaFold3 may facilitate FLS2 engineering, identification of naturally occurring receptors with expanded recognition and predicting recognition capability. We evaluated the accuracy of AlphaFold3 for predicting FLS2–flg22–SERK3 binding as a proxy for recognition coupled with experimental data from this study and others. We modelled 219 FLS2–flg22–SERK3 complexes, encompassing 26 FLS2 homologues and 2–97 natural flg22 variants per receptor (Supplementary Data 1). Overall, the high-confidence FLS2–flg22–SERK3 complexes adopt conformations that are similar to the published experimental *Arabidopsis thaliana* FLS2 crystal structure. The binding pocket accommodating the C-terminal region of flg22 is conserved across different receptor–ligand pairs, whereas the N-terminal region of flg22 displays greater conformational diversity. This is probably because of the additional requirement for co-receptor binding that constrains the binding pocket for flg22’s C terminus (Supplementary Data 1). From these trimeric complex models, we extracted the interface predicted template modelling (ipTM) score and minimal predicted aligned error (PAE) for specific chain pairs: FLS2–flg22 and flg22–SERK3. These metrics, obtained from three independent modelling attempts, serve as predictive indicators for flg22 recognition.

**Fig. 5:**
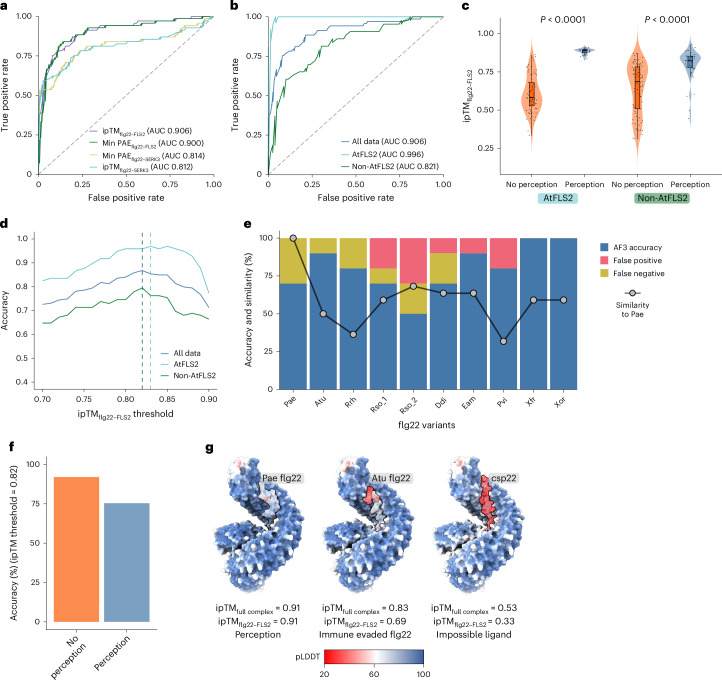
Identifying AlphaFold3 metrics for accurate prediction of flg22 recognition. **a**, ROC analysis of AlphaFold3. Four confidence metrics were separately analysed for 219 FLS2–flg22–SERK3 complexes: chain-pair average ipTM_flg22–FLS2_, ipTM_flg22–SERK3_, Minimal (Min) PAE_flg22–FLS2_ and Min PAE_flg22–SERK3_ from three independent modelling approaches. The diagonal dashed line represents a random classifier, and AUC values are annotated for each metric, indicating the model’s performance. **b**, ROC analyses using average ipTM_flg22–FLS2_ as the predictive metric. Three groups were analysed separately: all data (blue, *n* = 219), AtFLS2 only (cyan, *n* = 97) and non-AtFLS2 (green, *n* = 122). **c**, Average ipTM_flg22–FLS2_ for each FLS2–flg22–SERK3 complex whose perception has been validated experimentally (*n* = 219 combinations). Each dot represents the average ipTM_flg22–FLS2_ of three independent predictions extracted from each FLS2–flg22–SERK3 combination. Two-sided Wilcoxon rank-sum test was used to determine significance. The box in the boxplot represents the interquartile range (IQR), with the lower and upper hinges corresponding to the first and third quartiles, respectively. The whiskers extend to the smallest and largest values within 1.5× the IQR from the hinges, while the central line indicates the median. **d**, Accuracy with different ipTM_flg22–FLS2_ thresholds. Dashed vertical lines indicate the optimal threshold points for each group where accuracy is maximized with the ipTM threshold = 0.82 for all data and non-AtFLS2 data, and 0.83 for only AtFLS2 data. All data accuracy, 86.8%; AtFLS2 accuracy, 96.9%; non-AtFLS2 accuracy, 79.5%. **e**, Accuracy of AlphaFold3 for predicting immunogenicity of flg22 variants selected in this study (*n* = 10 receptors × 10 flg22 variants). Dots indicate the percentage similarity of each flg22 variant to the canonical Pae flg22. **f**, AlphaFold3 accuracy using a 0.82 ipTM_flg22–FLS2_ threshold for perception of flg22 ligands. **g**, AlphaFold3 models of AtFLS2 (*Arabidopsis thaliana*), AtBAK1 and flg22 from Pae that is perceived, the flg22 variant from Atu that is not perceived, and the impossible ligand csp22 from cold shock protein. High pLDDT indicates higher confidence and more accurate prediction.

**Extended Data Fig. 7:**
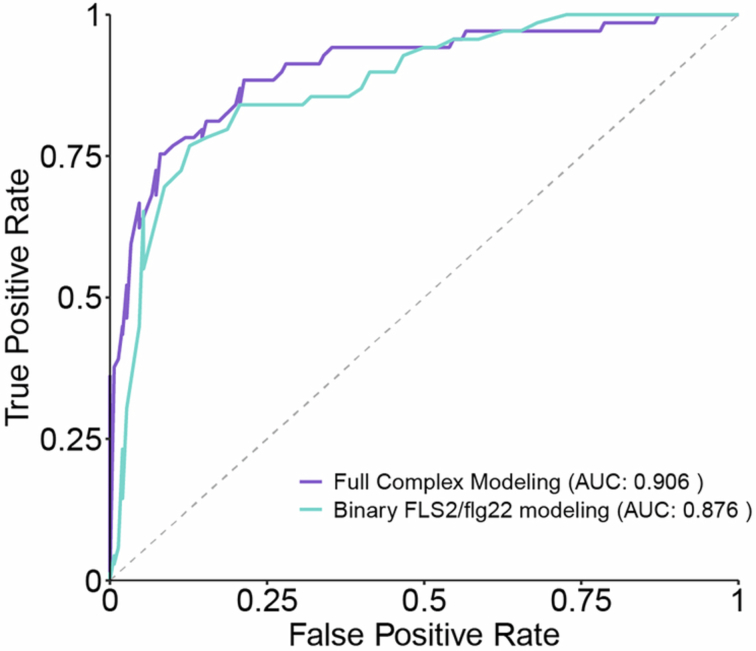
AlphaFold3 metrics from complete FLS2-flg22-SERK3 models provide better prediction. Receiver Operating Characteristic (ROC) curves comparing the predictive performance of two modeling approaches for flg22–FLS2 interactions, using ipTM_flg22-FLS2_: Full complex modeling (FLS2-flg22-SERK3, purple) and binary FLS2-flg22 modeling (teal). The dashed gray diagonal represents random predictions. The area under each ROC curve (AUC) is shown in the legend.

Receiver operating characteristic (ROC) analysis is commonly used to assess predictive model performance, with an area under the curve (AUC) value of 1 indicating complete accuracy^26^. Overall, AlphaFold3 can differentiate perception of flg22 through ipTM and Minimal PAE, but ipTM_flg22–FLS2_ achieved the best performance (AUC = 0.906) (Fig. 5a). Using ipTM_flg22–FLS2_, ROC analyses also indicate that AlphaFold3 has near perfect accuracy for predicting flg22 perception outcomes with AtFLS2, with an AUC value of 0.996 (Fig. 5b). AlphaFold3 exhibits moderate accuracy for predicting perception of other FLS2 homologues (AUC = 0.821) (Fig. 5b). High accuracy in predicting AtFLS2 recognition profiles is probably because of the availability of a solved FLS2 crystal structure from *Arabidopsis thaliana* bound to Pae flg22 (ref. ^8^), which provides more-precise structural constraints. Importantly, the presence of a structure also enables accurate perception of diverse ligands. To maximize overall accuracy, we established an optimal ipTM_flg22–FLS2_ threshold of 0.82 (Fig. 5c). When the ipTM_flg22–FLS2_ threshold was set to 0.82 for all data, AlphaFold3 achieves an overall accuracy rate of 86.8%, successfully predicting 92.0% of overall non-perceiving combinations and 75.4% of perceiving combinations (Fig. 5c,d). AlphaFold3 consistently positions the peptide ligand corresponding to the AtFLS2 region that interacts with flg22 (Fig. 5e). To investigate whether accuracy was impacted by flg22 variants, we analysed accuracy using the FLS2 homologues and ten flg22 variants investigated in this study (Fig. 5f). Notably, flg22 variants’ similarity to Pae flg22, which is included in the AtFLS2–flg22–BAK1 crystal structure, did not substantially impact prediction accuracy (Fig. 5f). We also investigated whether removing the co-receptor SERK3 from the modelling would affect predictive performance. Modelling the binary FLS2–flg22 complex slightly decreased performance compared to the full FLS2–flg22–SERK3 complex (AUC of 0.876 versus 0.906) (Extended Data Fig. 7). In summary, AlphaFold3 demonstrates promise in predicting recognition profiles of different FLS2 receptors.

### Diversifying selection on FLS2 concave-surface residues

**Fig. 6:**
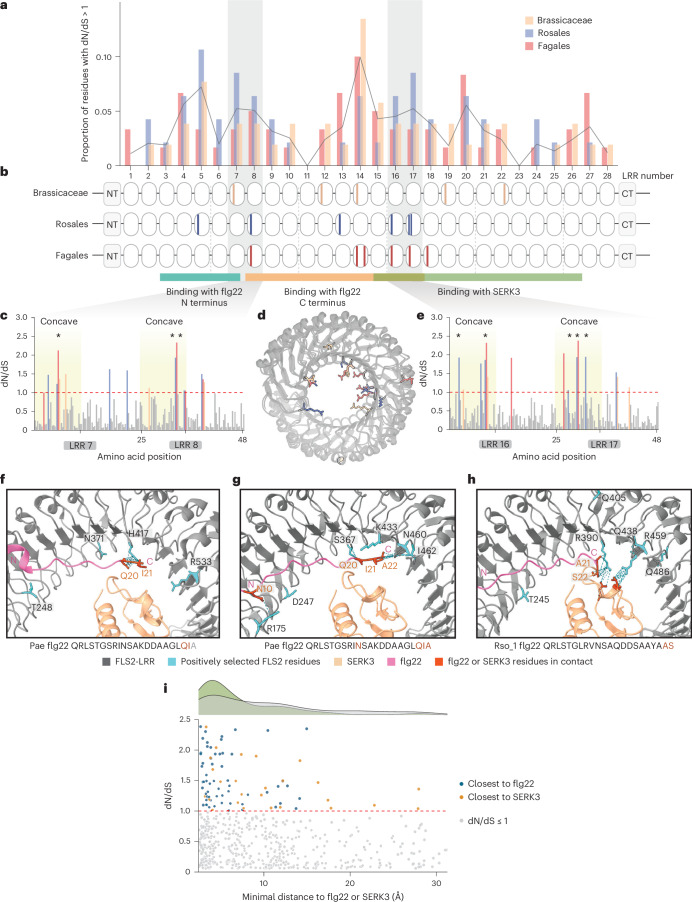
Evolutionary analysis of FLS2 from the Brassicaceae, Rosales and Fagales. **a**, Normalized proportion of FLS2 residues with dN/dS >1 across 28 LRR domains. The black line represents the average proportions from the three phylogenetic groups across each LRR domain. **b**, Schematic representations of FLS2 from three plant groups, with colour bars highlighting residues under positive selection with a BEB probability >0.95. **c**, dN/dS per codon for LRR 7 and 8. Residues with dN/dS >1 are highlighted with colour scheme representing Brassicaceae (peach), Rosales (slate) and Fagales (salmon). Concave-surface regions are shaded with light yellow. Residues under positive selection are marked with an asterisk (BEB probability >0.95). **d**, Overlay of the crystal structure of AtFLS2 (Protein Data Bank 4MN8) with AlphaFold3 models of MdFLS2 and QvFLS2, representing the three plant phylogenetic groups. Residues under positive selection (BEB probability >0.95) are highlighted using the colour scheme described in **b**. **e**, dN/dS per codon for LRR 16 and 17. Residues with dN/dS >1 are highlighted using the colour scheme described in **b**. Regions on the concave surface are shaded in light yellow. Residues under positive selection are marked with an asterisk (BEB probability >0.95). **f**–**h**, Structural visualization of the AtFLS2–BAK1–Paeflg22 (Protein Data Bank 4MN8) complex (**f**) alongside AlphaFold3 models of MdFLS2–NbSERK3A–Paeflg22 (**g**) and QvFLS2–NbSERK3A–Rso_1flg22 (**h**). FLS2 residues under positive selection are highlighted in cyan, whereas residues of the flg22 and SERK3 interacting with positively selected FLS2 residues (within a 5-Å distance) are shown in orange. The N and C termini of flg22 are labelled. **i**, dN/dS of each FLS2 concave-surface residue in relation to its minimal distance from flg22 or SERK3. Top, density distributions for residues (dN/dS >1, green; ≤1, grey). Bottom, scatter plot of all FLS2 concave-surface residues colour-coded by dN/dS and proximity to flg22 or SERK3. The dashed red line at dN/dS = 1 indicates the threshold between purifying and/or neutral and diversifying selection. dN/dS for each residue was calculated as described in **a**, then mapped onto the structural models shown in **f**–**h**. Minimal atom–atom distances between each FLS2 residue and flg22–SERK3 were extracted.

Plant immune receptors are subject to diversifying selection, driving functional divergence^27–30^. Broadened perception probably provides a selective advantage in pathogen recognition and relevant residues may be under positive selection. Therefore, we investigated whether residues in FLS2’s ectodomain are under positive selection. Three distinct phylogenetic groups comprising two plant orders and one family were selected: Fagales, Rosales and Brassicaceae. These groups were selected based on genomic resources and the presence of multiple genomes carrying a single copy of *FLS2* that is syntenic (Supplementary Fig. 1). Positive selection analysis was conducted using CodeML. Similar patterns of selection pressure were found across each group when scanning all 28 LRRs (Fig. 6a). Specifically, LRR 11 and LRR 23 were conserved (Fig. 6a). By contrast, residues with a higher ratio of non-synonymous to synonymous substitution rates (dN/dS) were predominantly located in LRRs 5–8 and LRRs 12–19 (Fig. 6a,b).

**Extended Data Fig. 8:**
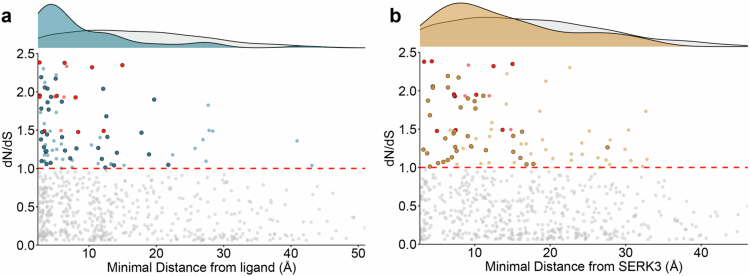
Positively selected residues on the FLS2 concave surface are located near the flg22 C-terminus or SERK3. **a**, dN/dS of each FLS2 concave-surface residue in relation to its minimal distance to flg22. The top part are the marginal density distributions of minimal distances for residues under positive selection (dN/dS > 1, blue) versus purifying/neutral selection (dN/dS ≤ 1, gray). The bottom part presents a scatter plot of all FLS2 concave-surface residues, color-coded by whether dN/dS ≤ 1 (gray) or dN/dS > 1 and closest to flg22 (blue). **b**, dN/dS of each FLS2 concave-surface residue in relation to its minimal distance to SERK3. The top part are the marginal density distributions of minimal distances for residues under positive selection (dN/dS > 1, gold) versus purifying/neutral selection (dN/dS ≤ 1, gray). The bottom part presents a scatter plot of all FLS2 concave-surface residues, color-coded by whether dN/dS ≤ 1 (gray) or dN/dS > 1 and closest to flg22 (gold). Residues with a high probability (BEB probability > 0.95) of being under positive selection are shown in red. LRRs 10–20, corresponding to the flg22 C-terminus and SERK3-binding region, are outlined in black. The dashed red line at dN/dS = 1 indicates the threshold between purifying/neutral and diversifying selection. Distances are shown in Å. dN/dS for each residue was calculated as described in (Fig. 6a), then mapped onto the structural models shown in (Fig. 6f–h). Minimal atom–atom distances between each FLS2 residue and flg22/SERK3 were extracted from the same structural models.

To identify specific residues under positive selection, the Bayes Empirical Bayes (BEB) method was used to calculate selection probabilities. Residues with a BEB probability exceeding 0.95 were considered positively selected. These residues are highlighted in Fig. 6b and mapped onto the structural models of AtFLS2, MdFLS2 (*Malus domestica*) and QvFLS2, representing the Brassicaceae, Rosales and Fagales, respectively (Fig. 6d). Of these positively selected residues, 88.2% were located on the concave surface of the LRR, mainly near the binding pockets for the flg22 polymorphic C terminus and the co-receptor (Fig. 6f–i and Extended Data Fig. 8). This aligns with previous findings that polymorphisms in the flg22 C terminus are key for canonical FLS2 immune evasion^15,17^ (Fig. 6c–e). The selection analyses mirror our experimental data for QvFLS2 and FLS2^XL^ engineering. Overall, our data suggest that integrating evolutionary analysis, protein modelling and amino acid properties is a promising strategy to identify receptors with expanded perception and pinpoint specific residues for engineering broader recognition.

## Discussion

Plants contain many PRRs, but MAMP diversity impairs receptor effectiveness. Using an interdisciplinary approach, we identified key residues along FLS2’s concave surface that enabled expanded recognition and engineered them into homologues with canonical perception. Our findings identify amino acid properties and AlphaFold3 metrics for predicting PRR recognition, providing a framework for engineering receptors to improve pathogen restriction.

Previous studies have demonstrated that exchanging sets of LRRs can alter ligand specificity of immune receptors^31–33^. Swapping LRRs 19–24 from SlFLS2 (S*. lycopersicum*) into AtFLS2 affected binding affinity to artificial flg22 variants^34^. In FLS2^XL^, LRR subdomain swaps with its close paralogue VrFLS2 highlighted the critical role of LRRs 12–18 in mediating Atu flg22 perception^15^. These data are consistent with our findings, which demonstrate the importance of specific residues in LRRs 10–20 for conferring expanded perception (Figs. 2 and 3). A recent preprint was able to transfer robust *Agrobacterium* flg22 perception from FLS2^XL^ to VvFLS2 (*Vitis vinifera*) using residue transfers from similar LRR regions (LRR 12–19)^35^. Previous efforts to create receptor variants with expanded recognition through site-directed random mutagenesis have slightly enhanced AtFLS2 sensitivity to polymorphic flg22 at micromolar concentrations^36^. We were able to pinpoint key residues crucial for ligand specificity by focusing on differences in the chemical properties of residues on the concave surface between receptors with different perception ranges (Figs. 2 and 3). A recent study focused on engineering the nucleotide-binding, leucine-rich repeat receptor (NLR) Sr50 to recognize a variant of the effector ligand AvrSr50, which evades immune detection^37^. Similarly, they showed that replacing residues on the LRR concave surface with those differing in charge or polarity can broaden the receptor’s recognition capabilities^37^. These findings suggest that targeting LRR concave-surface residues is a promising approach for engineering novel ligand perception across diverse LRR-containing immune receptors.

AlphaFold’s ability to predict protein–protein interactions has evolved rapidly. AlphaFold prediction of the NLR MLA3 (Mildew locus a 3) in complex with the fungal effector PWL2 (Pathogenicity toward Weeping Lovegrass 2), revealed that MLA3 shares a similar binding interface with other PWL2 targets^38^. Furthermore, this interface could be transferred to another NLR, resulting in an expanded effector recognition profile^38^. Compared with the cryo-electron microscopy structure, AlphaFold can predict the interactions between the critical Ser5 and Ser7 residues of the ligand SERINE-RICH ENDOGENOUS PEPTIDE 12 (SCOOP12) and the receptor *Arabidopsis* RK MIK2 (MALE DISCOVERER 1-INTERACTING RECEPTOR-LIKE KINASE 2), but is unable to completely predict the all residue interactions^39,40^. We used AlphaFold to pinpoint residues on the binding interface between FLS2 homologues and flg22 variants to engineer new recognition specificities (Figs. 2 and 3). Predictions involving AtFLS2, which has a known crystal structure, and various flg22 variants achieved a high accuracy of 96.9% (ipTM_flg22–FLS2_ threshold of 0.83). By contrast, complexes excluding AtFLS2–flg22 reached 79.5% accuracy (ipTM_flg22–FLS2_ threshold of 0.82). This indicates that obtaining additional crystal structures of receptor–ligand complexes will improve AlphaFold3’s accuracy to predict recognition. Future experimental investigations could determine if the ipTM thresholds for FLS2–flg22 can be broadly applied to other LRR-RKs.

Polymorphic ligand perception can occur through the functional diversification of a single receptor or the convergent evolution of a unique receptor with a distinct perception range. Beyond FLS2, some potato genotypes have homologues of the LRR-RK PERU (Pep-13 receptor unit) with expanded specificity against synthetic Pep-13 peptide variants^27^. Across two orders and one family, we detected similar patterns of diversifying selection on specific FLS2 LRRs, particularly those mapping to concave-surface residues (Fig. 6). One region undergoing diversifying selection maps to LRRs 12–19, aligning with our engineering of expanded ligand perception (Fig. 6). Overall, evidence suggests that expanded recognition specificity may be more common than previously thought.

Pathogens contain high evolutionary potential and can carry diverse MAMP epitopes that evade PRR perception^13,14,18^. Our findings reveal that all three FLS2 homologues with broadened perception recognize additional flg22 variants from important plant pathogens (Fig. 1). However, none have identical recognition profiles; it is unlikely that a single receptor could be engineered to recognize all flg22 variants, each may require distinct binding interfaces (Fig. 4).

This study highlights approaches to realize the potential for rational design of immune receptors by altering the binding interface. Coupling diversity analyses, protein modelling and biochemical parameters with experimental data can facilitate the identification and engineering of novel receptors with desired specificity for important pathogens. Narrowing specificity to a few amino acid residues is compatible with genome editing technologies that will expedite the deployment of the engineered resistance genes and reduce regulatory burdens^41–43^.

## Methods

### Plant materials and growth conditions

*N. benthamiana* plants were grown in a Conviron growth chamber at 26 °C with a 16 h light and 8 h dark photoperiod (180 μM m^−2^ s^−1^). Thirty-day-old (non-flowering) plants were used for *A. tumefaciens* mediated transient protein expression and subsequent immunity assays. The *N. benthamiana fls2-1/2* CRISPR–Cas9 mutant line was used for transient expression of FLS2 constructs and subsequent ROS and MAPK assays^16^.

### Selecting flg22 variants and peptide preparation

FliC sequences were collected from α-, β- and γ-proteobacterial genomes assessed in a previous study^18^. Protein sequences were aligned via MAFFT (v.7.310) using the following parameters: --reorder --thread 12 --maxiterate 1000 –auto^44^. A maximum-likelihood (ML) tree was built from the alignment using IQ-TREE2 (v.2.1.2) with the following parameters: -st AA -bb 1000 -m TEST -T AUTO^45^. The Newick file was visualized using the R packages phangorn (v.2.7.1), treeio (v.1.14.4) and ggtree (v.3.1.2.991). Epitope variants were selected from the previous study based on their abundance in the dataset, their diverse sequence in respect to the consensus flg22 epitope from *P. aeruginosa*, and unique C-terminal residues^18^.

Pae flg22 and Atu flg22 were synthesized by GenScript (≥95% purity) and Agrisera (≥95% purity), respectively. All other flg22 variants were synthesized by Shanghai Apeptide (≥95% purity). The peptides were solubilized in either water or dimethylsulfoxide, following the manufacturer’s recommendations. Details about the flg22 peptides can be found in Supplementary Table 2.

### Cloning of FLS2s

Plasmids harbouring the *FLS2*^*XL*^ and *VrFLS2* complementary DNA sequences were provided by G. Felix^15^. Plasmids containing the *TjFLS2* and *QvFLS2* genomic DNA sequences were supplied by Q. Cheng^16^. All full-length *FLS2s* were initially cloned into the pENTR/D-TOPO backbone (Invitrogen, K2400-20) and subsequently moved into the binary destination vector pGWB514 using the Gateway LR Clonase II enzyme mix (Invitrogen, 11791-100). The expression of *FLS2* constructs was driven by the 35S promoter and included a C-terminal HA tag. The synthetic FLS2 variants were reconstructed by assembling inserted regions that carry the amino acid changes into pENTR:FcFLS2 and pENTR:VrFLS2 using NEBuilder HiFi DNA Assembly (NEB, E5520). The details of all used constructs and primers are in Supplementary Tables 3 and 4.

### Transient expression of FLS2

*FLS2* variants were cloned into pGWB514 and transformed via electroporation into *A. tumefaciens* C58C1. The *N. benthamiana fls2-1/2* mutant was infiltrated with *A. tumefaciens* suspensions with an optical density at 600 nm of 0.6 induced with infiltration media (10 mM MgCl_2_, 5 mM MES-KOH (pH 5.6) and 0.2 mM acetosyringone) for 2 h. Twenty-four hours after infiltration, leaf disks were collected using a no. 1 or no. 5 cork borer (4 mm) for ROS assays and MAPK assay.

To visualize the expression of FLS2s, additional leaf disks were collected using a no. 7 cork borer at 24 hours post infiltration for protein extraction. The leaf disks were homogenized in 100 μl of Laemmli buffer and boiled for 5 min. Western blotting was conducted as described above and visualized with anti-HA–horseradish peroxidase antibody (1:3,000, Anti-HA-Peroxidase, High Affinity; Roche, 12013819001).

### ROS burst assay

Un-infiltrated wild-type *N. benthamiana* (containing native *NbFLS2*) and the *Nbfls2* CRISPR–Cas9 mutant transiently expressing *FLS2* were used for ROS assays. Leaf disks from test plants or infiltrated tissues were collected using a no. 1 cork borer (4 mm in diameter) and floated overnight in 200 μl of deionized distilled water in a Corning Costar 96-well White Solid Plate (Fisher, 07-200-589), covered with a plastic lid to minimize evaporation. The next day, the water was replaced with 100 μl of an assay solution containing flg22 variants. This solution consisted of 20 μM L-012 (a luminol derivative; Wako Chemicals, 120-04891), 10 mg ml^−1^ horseradish peroxidase (Sigma) and the flg22 variants. All treatments used 100 nM flg22 variants, with an equivalent amount of dimethylsulfoxide included for the control. Luminescence was measured using a BioTek Synergy H1 microplate reader (Agilent) with readings taken at 0.5-s intervals from each leaf disk over a period of 1 h.

For each plate, each treatment included 16 leaf disks from four individual plants. To calculate the average maximum relative luminescence units (RLUs) for each plant, the maximum RLU from the four leaf disks was determined and then averaged. These average maximum RLUs were normalized, with the average maximum RLU for water set to 0 and that for Pae flg22 set to 100,000. Finally, the normalized maximum RLUs for each flg22–FLS2 combination were averaged across all relevant plates. Normalization is conducted in Microsoft Excel.

### MAPK induction assay

For native *NbFLS2*, wild-type *N. benthamiana* leaves were infiltrated with *A. tumefaciens* C58C1 carrying a pGWB514 empty vector. For other *FLS2s*, *N. benthamiana fls2-1/2* mutant leaves were infiltrated with constructs as described above.

Twenty-four hours after infiltration, three leaf disks from the infiltrated areas were collected using a no. 5 cork borer (9 mm in diameter) and placed overnight in 1 ml of deionized water in a 24-well tissue culture plate (VWR, 10062-896), covered with a plastic lid to minimize evaporation. The following day, the water was replaced with 900 μl of either water or water containing 100 nM flg22 variants. Leaf disks from each well were then collected individually at 0 and 15 min after treatment, quickly frozen in liquid nitrogen and ground using pestles attached to an electric grinder (Conos AC-18S electric torque screwdriver). Protein extraction and MAPK assay-specific western blotting was conducted as previously described^10^. Anti-p44/42 MPK antibody (1:2,000, Cell Signaling Technology, 4370L) and goat anti-rabbit HRP secondary antibody (1:3,000, Bio-Rad, 170-5046) were used for visualization.

### Identification and evolutionary analysis of *FLS2* homologues

*FLS2* homologues were identified from available plant genomes in Brassicaceae, Rosales and Fagales orders using standard BLASTN–BLASTP search in the National Center for Biotechnology and Information (NCBI) database and Phytozome. Candidate sequences were removed if they were duplicated genes, pseudogenes, possessing major sequence gaps or incomplete FLS2 domain architecture. Sequence identities for all *FLS2* homologues are provided in Supplementary Data 2.

Full-length FLS2 protein sequences were initially aligned using MAFFT v.7.490. A ML tree was built from the alignment using PhyML v.3.3.2 with 1,000 bootstrap replicates^46^, using the best fit model selected by the Bayesian Information Criterion. Output trees were visualized on iTOL^47^.

To perform positive selection analyses, DNA sequences of *FLS2* LRR regions in the Brassicaceae, Rosales and Fagales were aligned using MAFFT v.7.490. Subsequently, ML trees of full-length FLS2 proteins and the aligned *FLS2* LRR region DNA sequences from each group, were used for positive selection analysis under site-specific models using the CodeML package (AAML in paml v.4.8a), part of the ETE Toolkit (v.3.1.3)^48,49^. After likelihood ratio test and BEB calculations, the posterior mean dN/dS value per codon and positively selected residues (BEB posterior probability >0.95) were extracted from M8.

To perform synteny analyses, selected annotated genomes from Brassicaceae, Fagales and Rosales were downloaded from the NCBI database. After manually curating the annotations, MCscan from the JCVI suite (https://github.com/tanghaibao/jcvi) was used to infer the synteny blocks in each group via all-against-all LAST^50^.

### Repeat conservation mapping

LRR domains of FLS2 proteins from the Vitales and Fagales orders were analysed. Repeat conservation scores were calculated using the standalone version of the RCM tool^20^. The concave surface is defined as the ‘xLxxLxLxxNx’ region of each LRR.

### Analyses of residue properties on the concave surface

Concave-surface-exposed residues are defined as the variable residues in the ‘xLxxLxLxxNx’ region of each LRR. For each FLS2 homologue analysed in this study, a global value of concave-surface-exposed residues was calculated across 44 different amino acid properties and scales using the R packages Peptides (v.2.4.5) and alakazam (v.1.3.0)^22^. Using the R packages FactoMineR (v.2.11) and factoextra (v.1.0.7), PCA was conducted on the matrix and dimensions 1 to 5 were visualized in the corrplot (v.0.92) package. To illustrate the impact of top correlated residue properties with possible ligand perception specificity, the bulkiness and charge (at pH 5.4) values were calculated along the surface-exposed residues for two canonical, two expanded and two synthetic variants, and plotted as a heatmap using ComplexHeatmap (v.2.6.2)^51^. Finally, FLS2 homologues of interest were modelled in ChimeraX with residue coloured by either bulkiness or charge values.

### Structural modelling and visualization

For FLS2 engineering, the protein complex of the LRR domain of FLS2 homologues with co-receptor NbSERK3A and flg22 variants were predicted through AlphaFold3 (ref. ^52^). Models with ipTM values >0.8 were saved for further analysis. FLS2 residues that are within 5 Å of the flg22 ligand and the co-receptor SERK3 were designated as candidate interface residues to capture potential atomic contact.

For benchmarking AlphaFold3’s flg22 perception prediction, protein sequences of flg22 variants, selected FLS2–LRRs and compatible SERK3–LRRs were input into AlphaFold3 Server to model the different combinations of FLS2–flg22–SERK3 complex^52^. Each combination was modelled three times independently. After validating the AlphaFold3 result with experimental data, the best ipTM threshold value to achieve the highest accuracy for AF3-based prediction is 0.82. All protein structures were visualized and analysed in ChimeraX (v.1.8) and PyMOL (v.2.5.2).

### Reporting summary

Further information on research design is available in the Nature Portfolio Reporting Summary linked to this article.

## Supplementary information

Supplementary Information Supplementary Fig. 1.

Reporting Summary

Supplementary Data 1 AlphaFold3 based prediction of flg22 recognition.

Supplementary Data 2 FLS2 homologs identified for evolutionary analysis.

Supplementary Tables 1–4 Primers, constructs and peptides used in the study, and amino acid substitution details.

## Source data

Source Data Figs. 1–3 and Extended Data Figs. 2 and 3 Unprocessed and uncropped western blots.

## Data Availability

All raw data underlying Figs. 1–6 and Extended Data Figs. 1–8 are available via Zenodo at 10.5281/zenodo.13738225 (ref. ^53^). Accession numbers of FLS2 and flg22 sequences in this study are derived from NCBI and provided in Supplementary Data 2, and are available via GitHub at https://github.com/jerrytli/FLS2_engineering. All plasmids generated in this study were deposited to Addgene (226412–226420) for public distribution. Source data are provided with this paper.

## Code availability

R scripts used for data analysis and plotting are accessible via GitHub at https://github.com/jerrytli/FLS2_engineering.
