# Supplementary material for: Unlocking expanded flagellin perception through rational receptor engineering
Source: Nat Plants. 2025 Jul 28;11(8):1628–41. doi: 10.1038/s41477-025-02049-y (PMC12364713; doi:10.1038/s41477-025-02049-y)
Supplement: Supplementary file 1 — Supplementary Fig. 1. [file 41477_2025_2049_MOESM1_ESM.pdf]

nature plants

Supplementary information https://doi.org/10.1038/s41477-025-02049-y

Unlocking expanded flagellin perception through rational receptor engineering

In the format provided by the authors and unedited

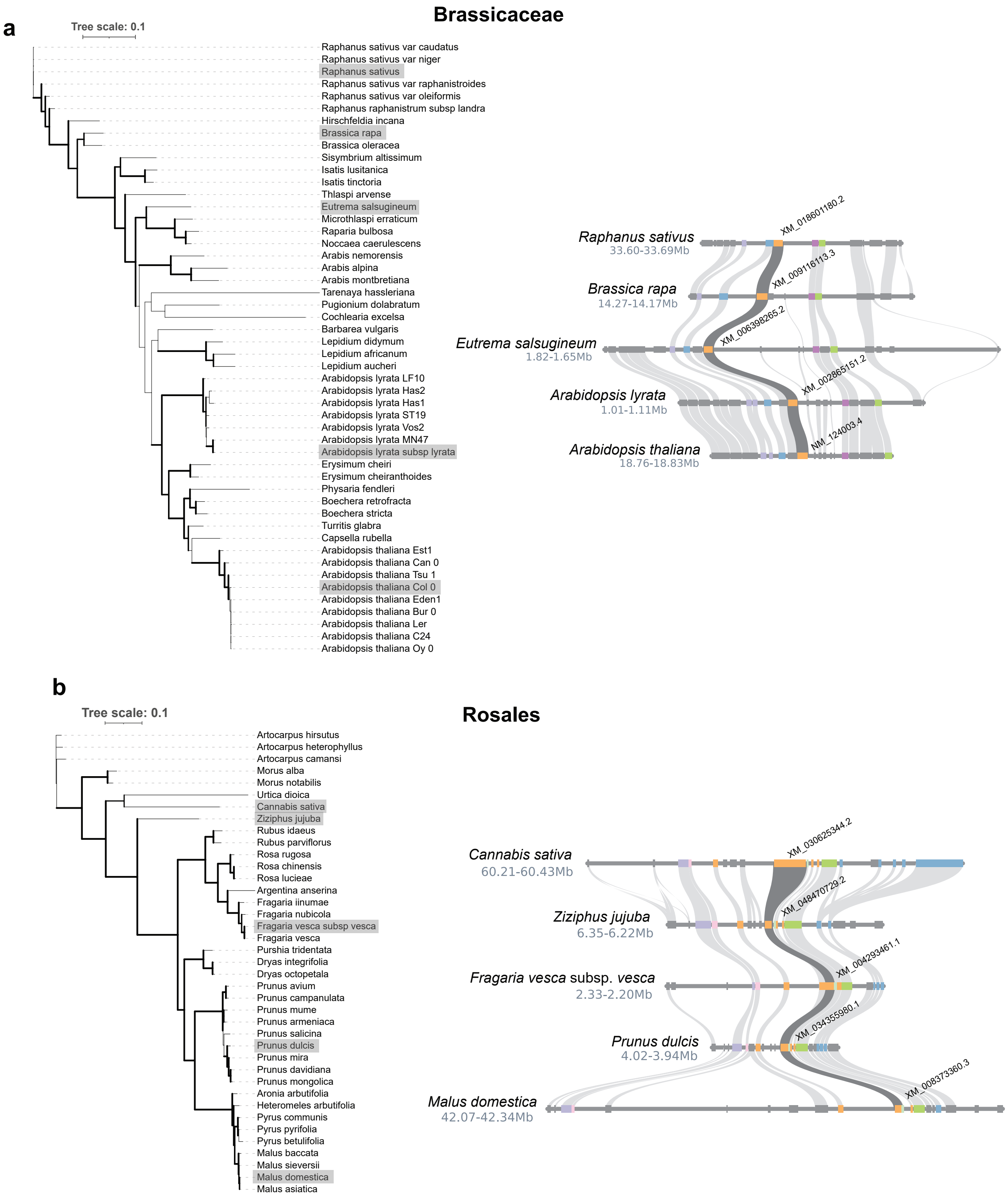
a
Brassicaceae
Tree scale: 0.1
Raphanus sativus var caudatus
Raphanus sativus var niger
Raphanus sativus
Raphanus sativus var raphanistroides
Raphanus sativus var oleiformis
Raphanus raphanistrum subsp landra
Hirschfeldia incana
Brassica rapa
Brassica oleracea
Sisymbrium altissimum
Isatis lusitanica
Isatis tinctoria
Thlaspi arvense
Eutrema salsugineum
Microthlaspi erraticum
Raparia bulbosa
Noccaea caerulescens
Arabis nemorensis
Arabis alpina
Arabis montbretiana
Tarenaya hassleriana
Pugionium dolabratum
Cochlearia excelsa
Barbarea vulgaris
Lepidium didymum
Lepidium africanum
Lepidium aucheri
Arabidopsis lyrata LF10
Arabidopsis lyrata Has2
Arabidopsis lyrata Has1
Arabidopsis lyrata ST19
Arabidopsis lyrata Vos2
Arabidopsis lyrata MN47
Arabidopsis lyrata subsp lyrata
Erysimum cheiri
Erysimum cheiranthoides
Physaria fendleri
Boechera retrofracta
Boechera stricta
Turritis glabra
Capsella rubella
Arabidopsis thaliana Est1
Arabidopsis thaliana Can 0
Arabidopsis thaliana Tsu 1
Arabidopsis thaliana Col 0
Arabidopsis thaliana Eden1
Arabidopsis thaliana Bur 0
Arabidopsis thaliana Ler
Arabidopsis thaliana C24
Arabidopsis thaliana Oy 0
Raphanus sativus
33.60-33.69Mb
XM_018601180.2
Brassica rapa
14.27-14.17Mb
XM_009116113.3
Eutrema salsugineum
1.82-1.65Mb
XM_006398265.2
Arabidopsis lyrata
1.01-1.11Mb
XM_002865151.2
Arabidopsis thaliana
18.76-18.83Mb
NM_124003.4
b
Rosales
Tree scale: 0.1
Artocarpus hirsutus
Artocarpus heterophyllus
Artocarpus camansi
Morus alba
Morus notabilis
Urtica dioica
Cannabis sativa
Ziziphus jujuba
Rubus idaeus
Rubus parviflorus
Rosa rugosa
Rosa chinensis
Rosa lucieae
Argentina anserina
Fragaria iinumae
Fragaria nubicola
Fragaria vesca subsp vesca
Fragaria vesca
Purshia tridentata
Dryas integrifolia
Dryas octopetala
Prunus avium
Prunus campanulata
Prunus mume
Prunus armeniaca
Prunus salicina
Prunus dulcis
Prunus mira
Prunus davidiana
Prunus mongolica
Aronia arbutifolia
Heteromeles arbutifolia
Pyrus communis
Pyrus pyrifolia
Pyrus betulifolia
Malus baccata
Malus sieversii
Malus domestica
Malus asiatica
Cannabis sativa
60.21-60.43Mb
XM_030625344.2
Ziziphus jujuba
6.35-6.22Mb
XM_048470729.2
Fragaria vesca subsp. vesca
2.33-2.20Mb
XM_004293461.1
Prunus dulcis
4.02-3.94Mb
XM_034355980.1
Malus domestica
42.07-42.34Mb
XM_008373360.3

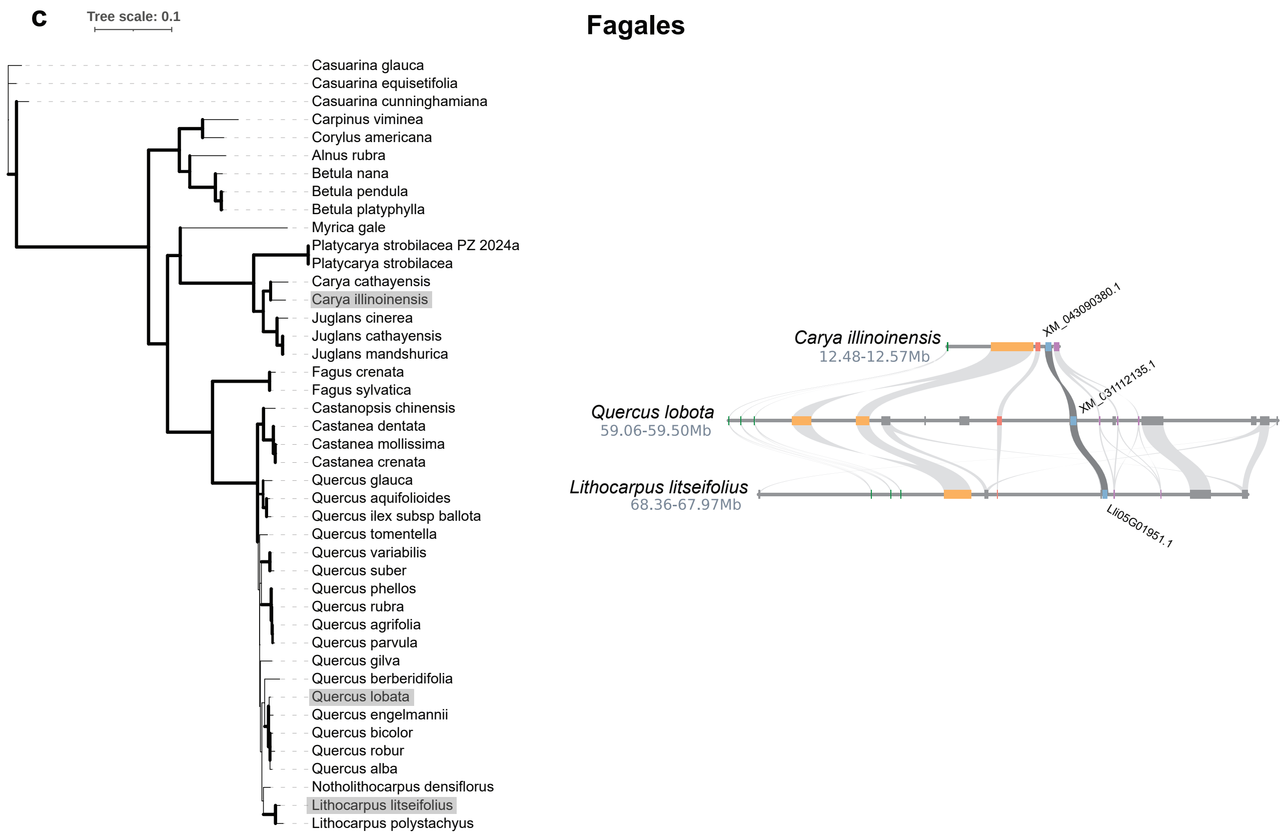

Supplementary Figure 1: Phylogenetic and microsynteny analysis of FLS2 in Brassicaceae, Rosales and Fagales. a to c, Brassicaceae, Rosales and Fagales. **Left**: Maximum likelihood phylogeny constructed using single-copy, full-length FLS2 protein sequences from each phylogenetic group with 1000 bootstrap repeats. Branches supported by > 70% bootstrap values are indicated with thick dark lines. Species included in the microsynteny analysis are shaded gray. **Right**: Microsynteny analysis of FLS2 alongside ten upstream and downstream genes. Orthologous gene pairs are linked by light gray bands. FLS2 pairs are emphasized with dark gray bands, with NCBI identifiers labeled on the side. Only genomes with high-quality annotations are included in this analysis.
