# Supplementary material for: Unlocking expanded flagellin perception through rational receptor engineering
Source: Nat Plants. 2025 Jul 28;11(8):1628–41. doi: 10.1038/s41477-025-02049-y (PMC12364713; doi:10.1038/s41477-025-02049-y)
Supplement: Supplementary file 2 — Reporting Summary [file 41477_2025_2049_MOESM2_ESM.pdf]

nature portfolio

Corresponding author(s): Gitta Coaker

Last updated by author(s): Feb 18, 2025

Reporting Summary

Nature Portfolio wishes to improve the reproducibility of the work that we publish. This form provides structure for consistency and transparency in reporting. For further information on Nature Portfolio policies, see our Editorial Policies and the Editorial Policy Checklist.

Statistics

For all statistical analyses, confirm that the following items are present in the figure legend, table legend, main text, or Methods section.

n/a	Confirmed	
☐	☒	The exact sample size (*n*) for each experimental group/condition, given as a discrete number and unit of measurement
☐	☒	A statement on whether measurements were taken from distinct samples or whether the same sample was measured repeatedly
☐	☒	The statistical test(s) used AND whether they are one- or two-sided<br>*Only common tests should be described solely by name; describe more complex techniques in the Methods section.*
☒	☐	A description of all covariates tested
☐	☒	A description of any assumptions or corrections, such as tests of normality and adjustment for multiple comparisons
☐	☒	A full description of the statistical parameters including central tendency (e.g. means) or other basic estimates (e.g. regression coefficient) AND variation (e.g. standard deviation) or associated estimates of uncertainty (e.g. confidence intervals)
☐	☒	For null hypothesis testing, the test statistic (e.g. *F*, *t*, *r*) with confidence intervals, effect sizes, degrees of freedom and *P* value noted<br>*Give P values as exact values whenever suitable.*
☒	☐	For Bayesian analysis, information on the choice of priors and Markov chain Monte Carlo settings
☒	☐	For hierarchical and complex designs, identification of the appropriate level for tests and full reporting of outcomes
☒	☐	Estimates of effect sizes (e.g. Cohen's *d*, Pearson's *r*), indicating how they were calculated

Our web collection on statistics for biologists contains articles on many of the points above.

Software and code

Policy information about availability of computer code

Data collection	ROS assay data were collected from BioTek Synergy H1 microplate reader (Agilent).<br>Western blot data were collected by using SuperSignal™ West Pico PLUS Chemiluminescent Substrate (ThermoFisher Scientific #34580) and visualized by ChemiDoc™ Touch Gel Imaging System (Bio-Rad).

Data analysis	ROS normalization is conducted using Microsoft Excel.<br>Quantified ROS and MAPK assay data is analyzed using GraphPad Prism 9, with one-way ANOVA and Dunnett's multiple comparison test on the non-scaled data to determine statistical significance between control (water) and treatment groups.<br>Sequence alignment is conducted using MAFFT (v7.310) for flg22 or MAFFT (v7.490) for FLS2.<br>Phylogeny making: IQ-TREE2 (v2.1.2) or PhyML 3.3.2<br>Phylogeny visualization: phangorn (v2.7.1), treeio (v1.14.4), and ggtree (v3.1.2.991) and iTOL online tool.<br>Micro-synteny analysis is conducted using JCVI suite (https://github.com/tanghaibao/jcvi) .<br>Positive selection analysis were conducted with ETE Toolkit v 3.1.3, using CodeML package (AAML in paml version 4.8a).<br>Repeat Conservation Mapping was conducted with the standalone version of the RCM tool.<br>Concave surface residue properties were analyzed using customized R script and packages: Peptides (v2.4.5) and alakazam (v1.3.0).<br>Principal Component Analysis: R packages FactoMineR (v2.11) and factoextra (v1.0.7). Heat map generated using ComplexHeatmap (v2.6.2)<br>Structural analysis was conducted using Chimera-X (v1.8) and PyMOL (2.5.2).<br>AlphaFold3 benchmarking data were analyzed using R packages pROC (1.18.5). Wilcoxon rank-sum test was use to determine significance for Figure 5c.<br>Other analysis involved with R scripts were provided in (https://github.com/jerrytli/FLS2_engineering)

For manuscripts utilizing custom algorithms or software that are central to the research but not yet described in published literature, software must be made available to editors and reviewers. We strongly encourage code deposition in a community repository (e.g. GitHub). See the Nature Portfolio guidelines for submitting code & software for further information.

Data

Policy information about availability of data

All manuscripts must include a data availability statement. This statement should provide the following information, where applicable:

- Accession codes, unique identifiers, or web links for publicly available datasets
- A description of any restrictions on data availability
- For clinical datasets or third party data, please ensure that the statement adheres to our policy

All raw data underlying each figure is available on Zenodo (doi:10.5281/zenodo.15180368). Accession numbers of flg22 and FLS2 sequences in this study are derived from NCBI and provided in (Supplementary Data 2) and GitHub Repository (https://github.com/jerrytli/FLS2_engineering). All plasmids generated in this study were deposited to Addgene (226412 - 226420) for public distribution.

Research involving human participants, their data, or biological material

Policy information about studies with human participants or human data. See also policy information about sex, gender (identity/presentation), and sexual orientation and race, ethnicity and racism.

Reporting on sex and gender	n/a
Reporting on race, ethnicity, or other socially relevant groupings	n/a
Population characteristics	n/a
Recruitment	n/a
Ethics oversight	n/a

Note that full information on the approval of the study protocol must also be provided in the manuscript.

Field-specific reporting

Please select the one below that is the best fit for your research. If you are not sure, read the appropriate sections before making your selection.

☒ Life sciences ☐ Behavioural & social sciences ☐ Ecological, evolutionary & environmental sciences

For a reference copy of the document with all sections, see nature.com/documents/nr-reporting-summary-flat.pdf

Life sciences study design

All studies must disclose on these points even when the disclosure is negative.

Sample size	Sample size described for ROS assays were chosen according to previously established standards including D.M. Stevens et al, (https://doi.org/10.1073/pnas.2319499121) , Trinh et al, (https://doi.org/10.1093/plphys/kiad263). Each ROS assays use 16 leaf disks from 4 individual plants for each treatment.
Data exclusions	No data were excluded.

Replication	All experiments were performed with at least three biological replicates and were independently repeated twice with similar results.
Randomization	Nicotiana benthamiana plants were grown under the same condition in the same growth chamber and randomly selected for ROS and MAPK assays. For each assay, Leaf disks that harboring designated receptors were randomly extracted from testing plants for control and different peptide treatment.
Blinding	Investigators were not blinded to group allocation during the experiments, but the data collection and assessment were repeated by multiple independent experiments and independent researchers (when possible) to ensure that the analyses are as objective as possible.

Reporting for specific materials, systems and methods

We require information from authors about some types of materials, experimental systems and methods used in many studies. Here, indicate whether each material, system or method listed is relevant to your study. If you are not sure if a list item applies to your research, read the appropriate section before selecting a response.

Materials & experimental systems

n/a	Involved in the study	
☐	☒	Antibodies
☒	☐	Eukaryotic cell lines
☒	☐	Palaeontology and archaeology
☒	☐	Animals and other organisms
☒	☐	Clinical data
☒	☐	Dual use research of concern
☐	☒	Plants

Methods

n/a	Involved in the study	
☒	☐	ChIP-seq
☒	☐	Flow cytometry
☒	☐	MRI-based neuroimaging

Antibodies

Antibodies used	anti HA-HRP antibody (Roche, #12013819001, Anti-HA-Peroxidase, High Affinity; 1:3000)<br>anti-p44/42 MPK antibody (1:2,000, Cell Signaling Technology #4370L)<br>Goat anti-rabbit HRP secondary antibody (1:3,000, Bio-Rad #170-5046)
Validation	anti HA-HRP antibody (Roche, #12013819001, Anti-HA-Peroxidase, High Affinity; 1:3000) :https://www.sigmaaldrich.com/US/en/product/roche/12013819001?srsltid=AfmBOoo5XzjgTTM0VhiqCHOpCYkNuaCAR0e9vx6cnSyuYR3YuYYlayZj&icid=sharepdp-clipboard-copy-productdetailpage<br><br>anti-p44/42 MPK antibody (1:2,000, Cell Signaling Technology #4370L): https://www.cellsignal.com/products/primary-antibodies/phospho-p44-42-mapk-erk1-2-thr202-tyr204-d13-14-4e-xp-rabbit-mab/4370?srsltid=AfmBOorg9CQtyPfdm-9XNdbbajFDlpatAj3wFLFb7CFjmoaJYcBhiY8a

Dual use research of concern

Policy information about dual use research of concern

Hazards

Could the accidental, deliberate or reckless misuse of agents or technologies generated in the work, or the application of information presented in the manuscript, pose a threat to:

No	Yes	
☒	☐	Public health
☒	☐	National security
☒	☐	Crops and/or livestock
☒	☐	Ecosystems
☒	☐	Any other significant area

Experiments of concern

Does the work involve any of these experiments of concern:

No	Yes	
☒	☐	Demonstrate how to render a vaccine ineffective
☒	☐	Confer resistance to therapeutically useful antibiotics or antiviral agents
☒	☐	Enhance the virulence of a pathogen or render a nonpathogen virulent
☒	☐	Increase transmissibility of a pathogen
☒	☐	Alter the host range of a pathogen
☒	☐	Enable evasion of diagnostic/detection modalities
☒	☐	Enable the weaponization of a biological agent or toxin
☒	☐	Any other potentially harmful combination of experiments and agents

Plants

Seed stocks	Wild-type Nicotiana benthamiana, Nicotiana benthamiana CRISPR/Cas9 fls2 mutant
Novel plant genotypes	Nicotiana benthamiana CRISPR/Cas9 fls2 mutant were generated by CRISPR/Cas9 based gene editing targeting NbFLS2-1 and NbFLS2-2. The mutant line is provided by outside research group.
Authentication	The Nicotiana benthamiana CRISPR/Cas9 fls2 mutant line were verified in the lab via PCR and subsequent Sanger sequencing. Mutant phenotypes were verified by challenging the mutant leaf disks with flg22 elicitor.
