# Supplementary material for: Unlocking expanded flagellin perception through rational receptor engineering
Source: Nat Plants. 2025 Jul 28;11(8):1628–41. doi: 10.1038/s41477-025-02049-y (PMC12364713; doi:10.1038/s41477-025-02049-y)

Figure 1

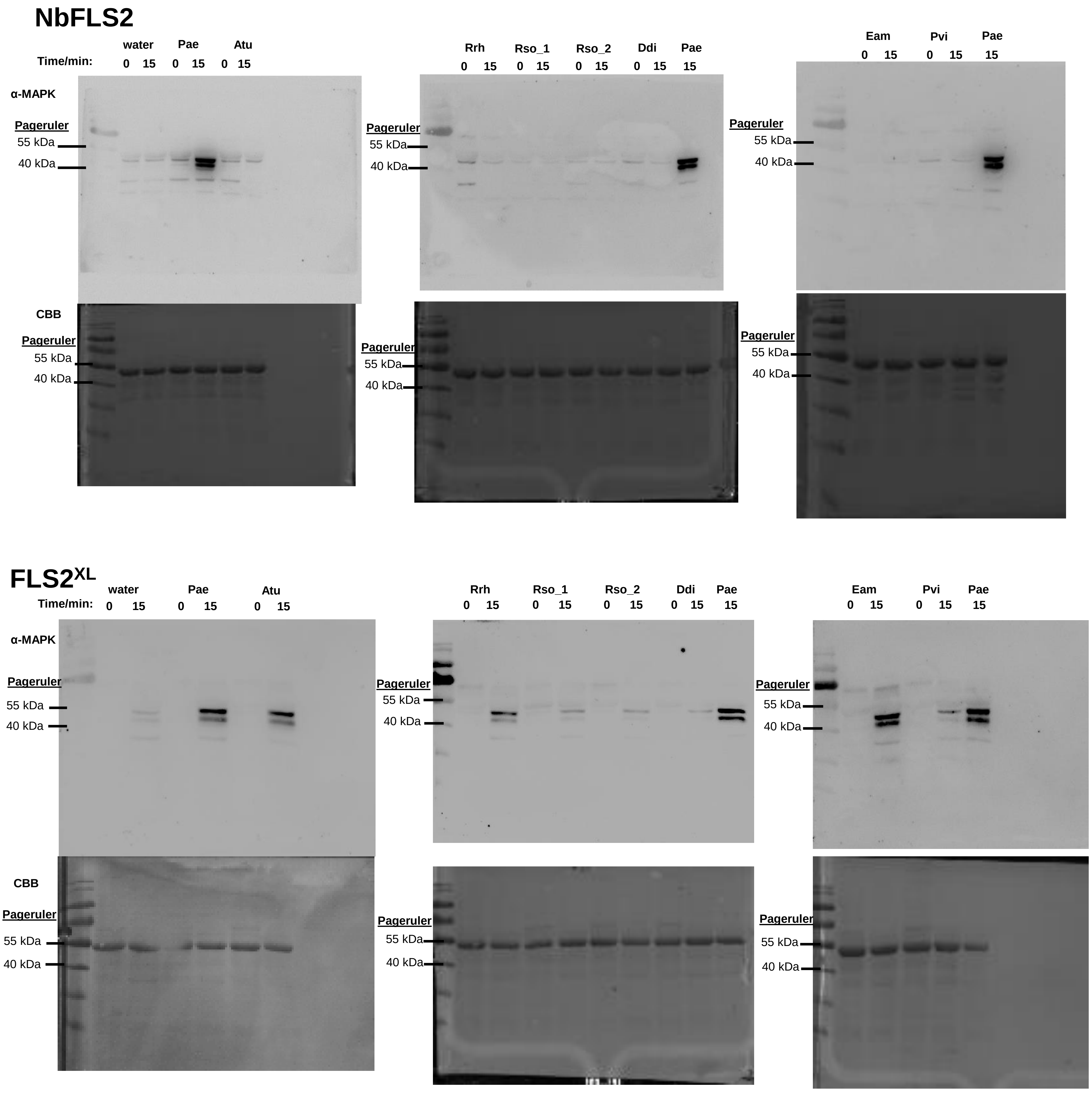

NbFLS2
water Pae Atu
Time/min: 0 15 0 15 0 15
α-MAPK
Pageruler
55 kDa
40 kDa
CBB
Rrh Rso_1 Rso_2 Ddi Pae
0 15 0 15 0 15 0 15 15
Eam Pvi Pae
0 15 0 15 15
FLS2XL
water Pae Atu
Time/min: 0 15 0 15 0 15
Rrh Rso_1 Rso_2 Ddi Pae
0 15 0 15 0 15 0 15 15
Eam Pvi Pae
0 15 0 15 15

Figure 1

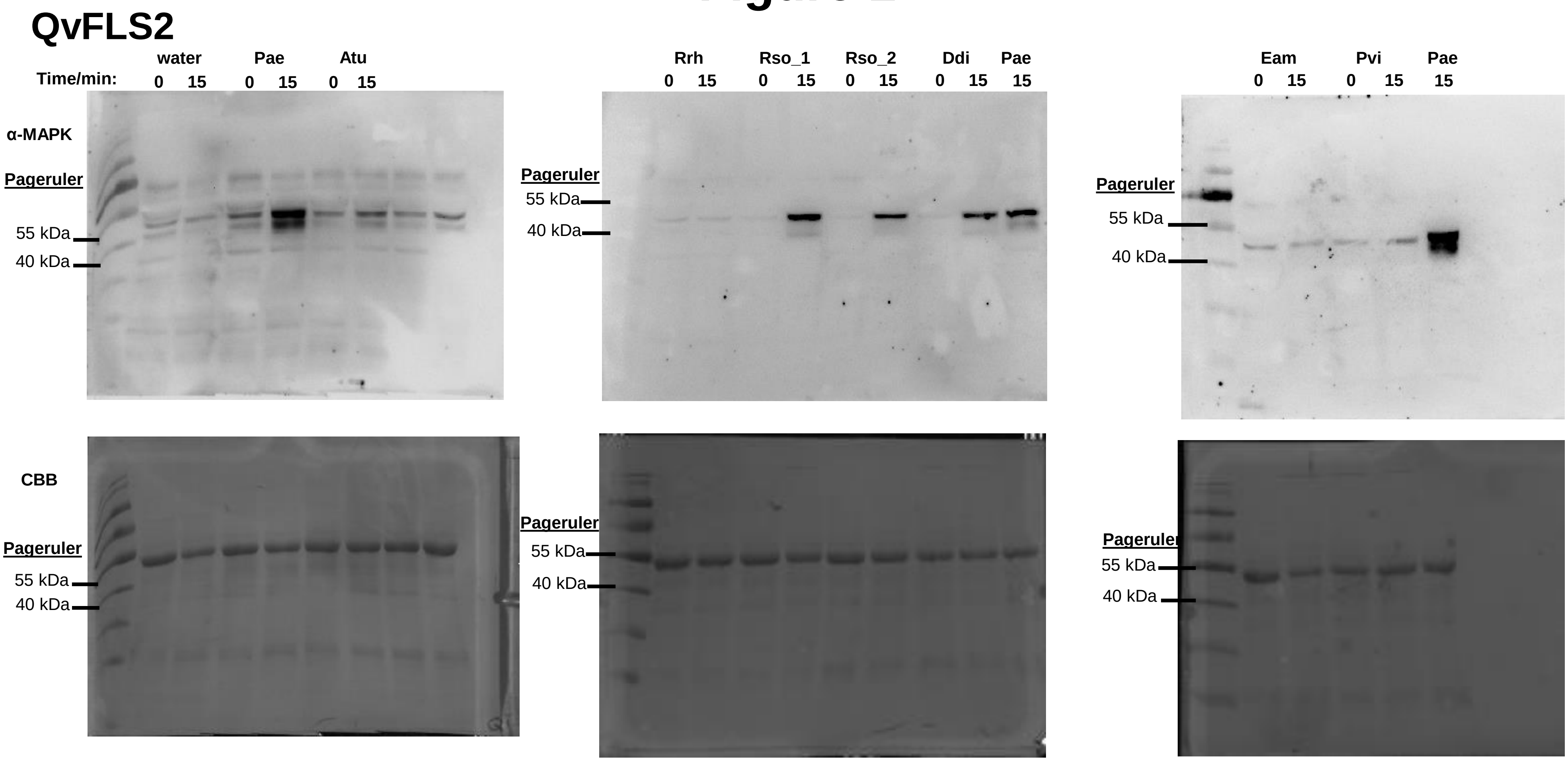
QvFLS2
water
Pae
Atu
Time/min:
0 15 0 15 0 15
α-MAPK
Pageruler
55 kDa
40 kDa
CBB
Pageruler
55 kDa
40 kDa
Rrh
Rso_1
Rso_2
Ddi
Pae
0 15 0 15 0 15 0 15 15
Pageruler
55 kDa
40 kDa
Pageruler
55 kDa
40 kDa
Eam
Pvi
Pae
0 15 0 15 15
Pageruler
55 kDa
40 kDa
Pageruler
55 kDa
40 kDa

Figure 2

QvFLS2

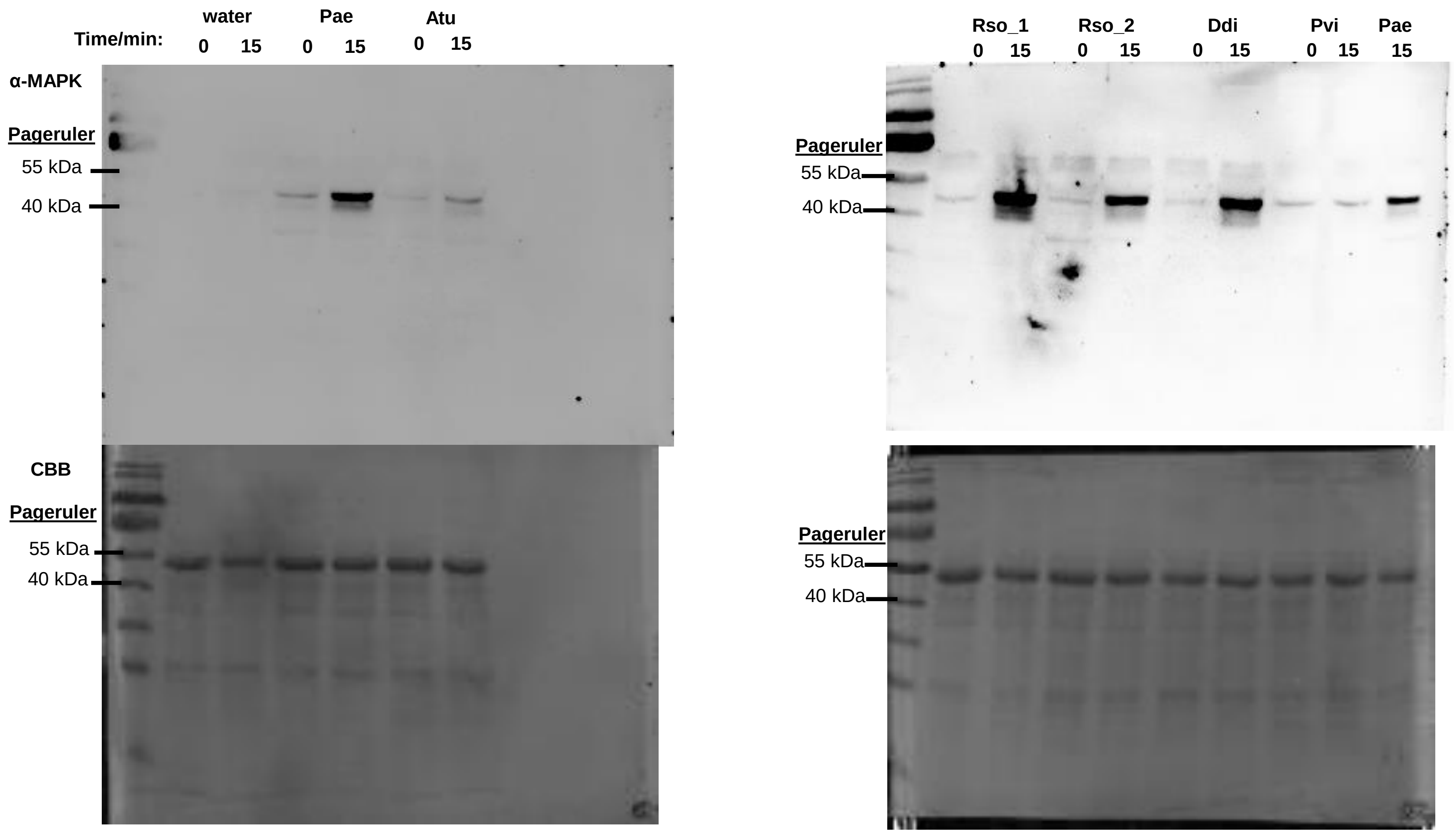

FcFLS2

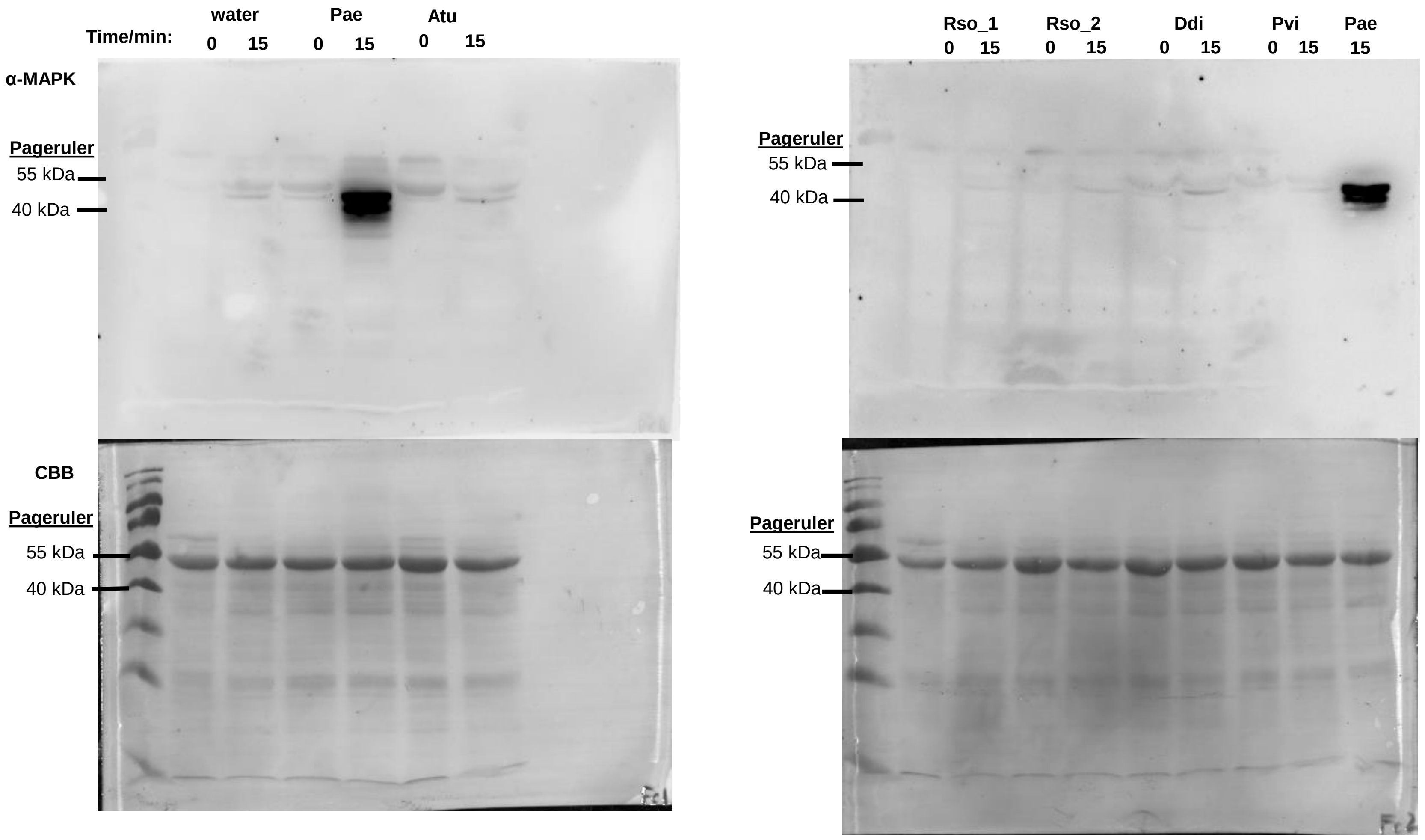

Figure 2

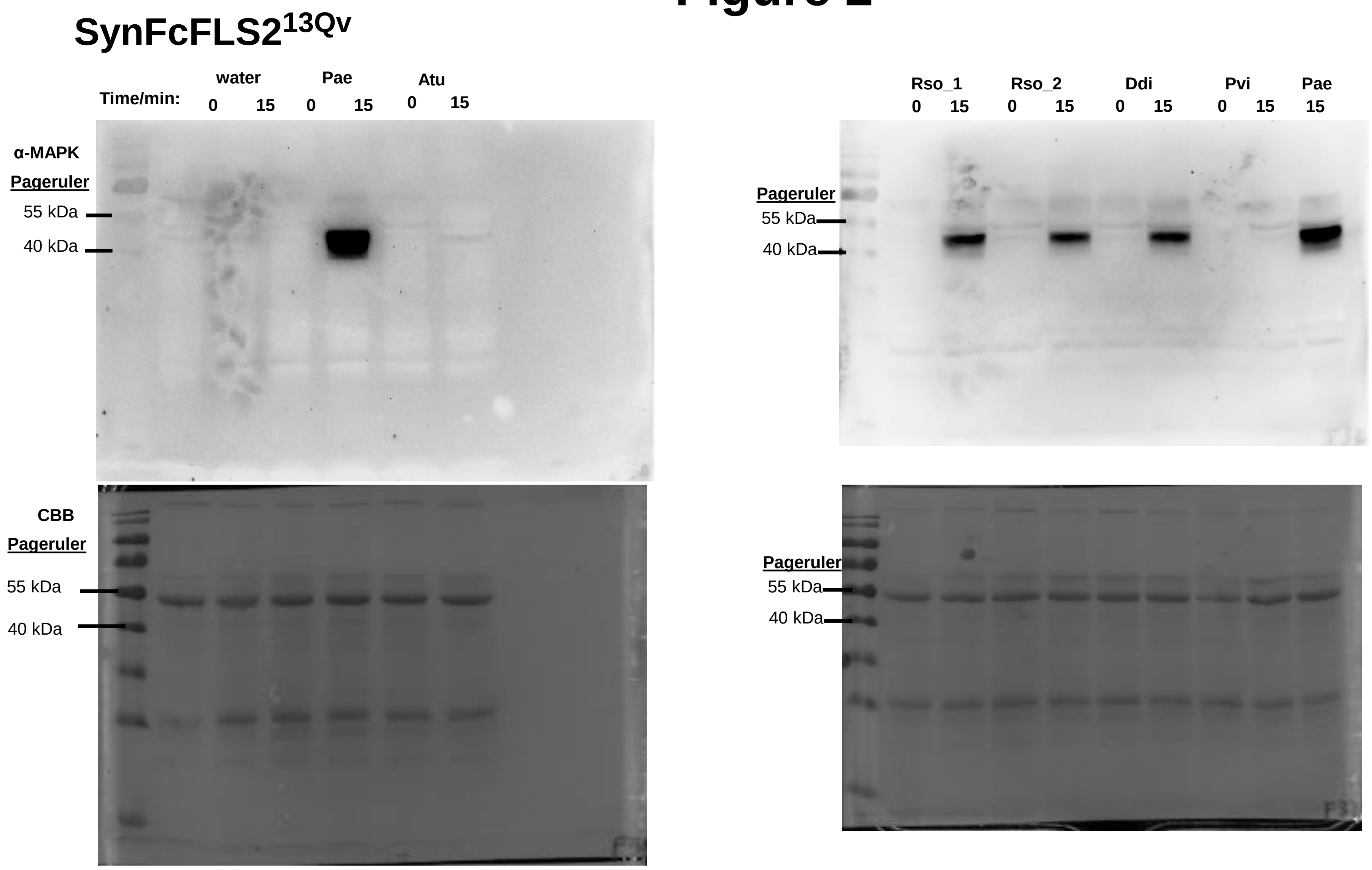
SynFcFLS2^{13Qv}
water
Pae
Atu
Time/min:
0 15 0 15 0 15
α-MAPK
Pageruler
55 kDa
40 kDa
CBB
Pageruler
55 kDa
40 kDa
Rso_1
Rso_2
Ddi
Pvi
Pae
0 15 0 15 0 15 0 15 15
Pageruler
55 kDa
40 kDa
Pageruler
55 kDa
40 kDa

Figure 3

FLS2^{XL}

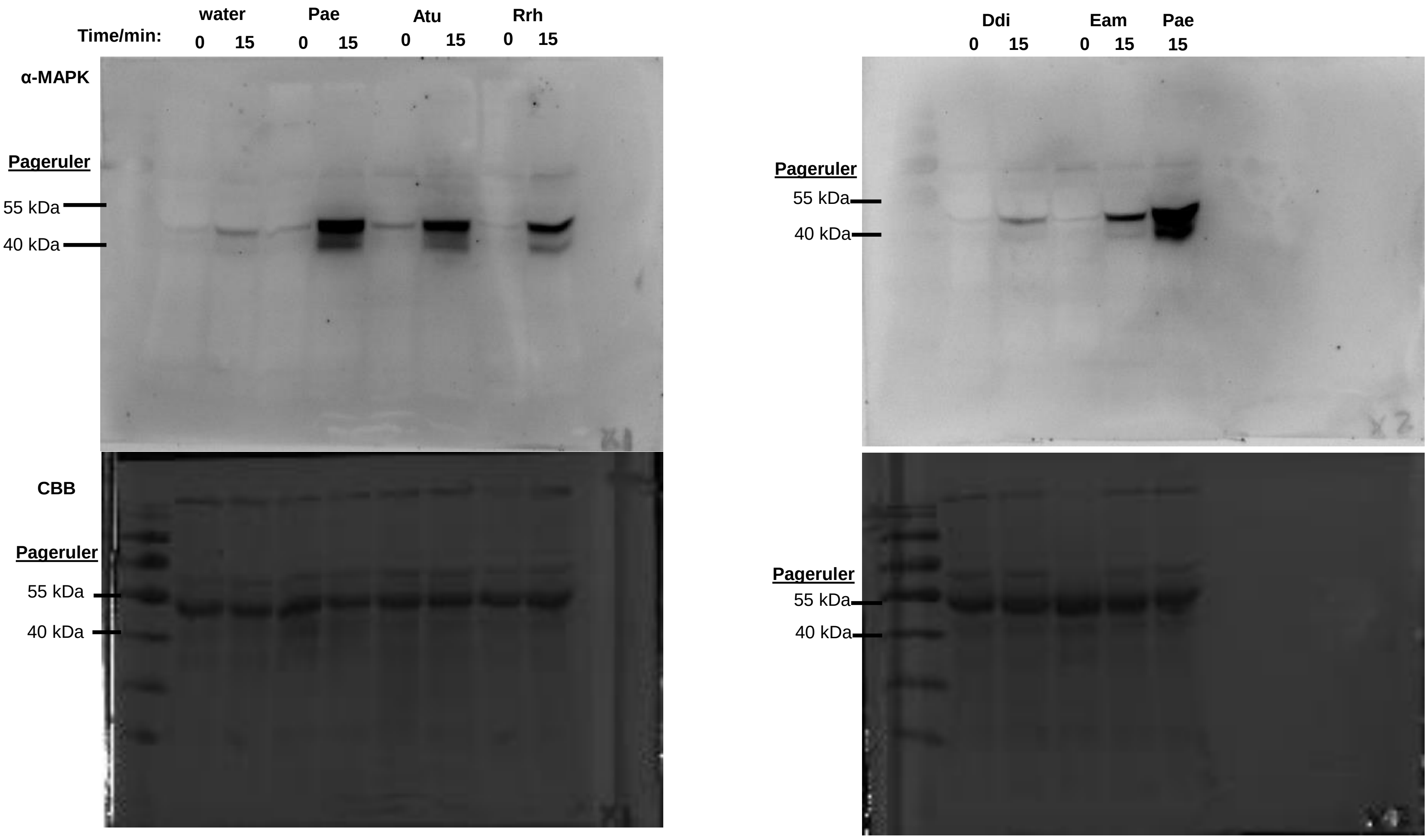

VrFLS2

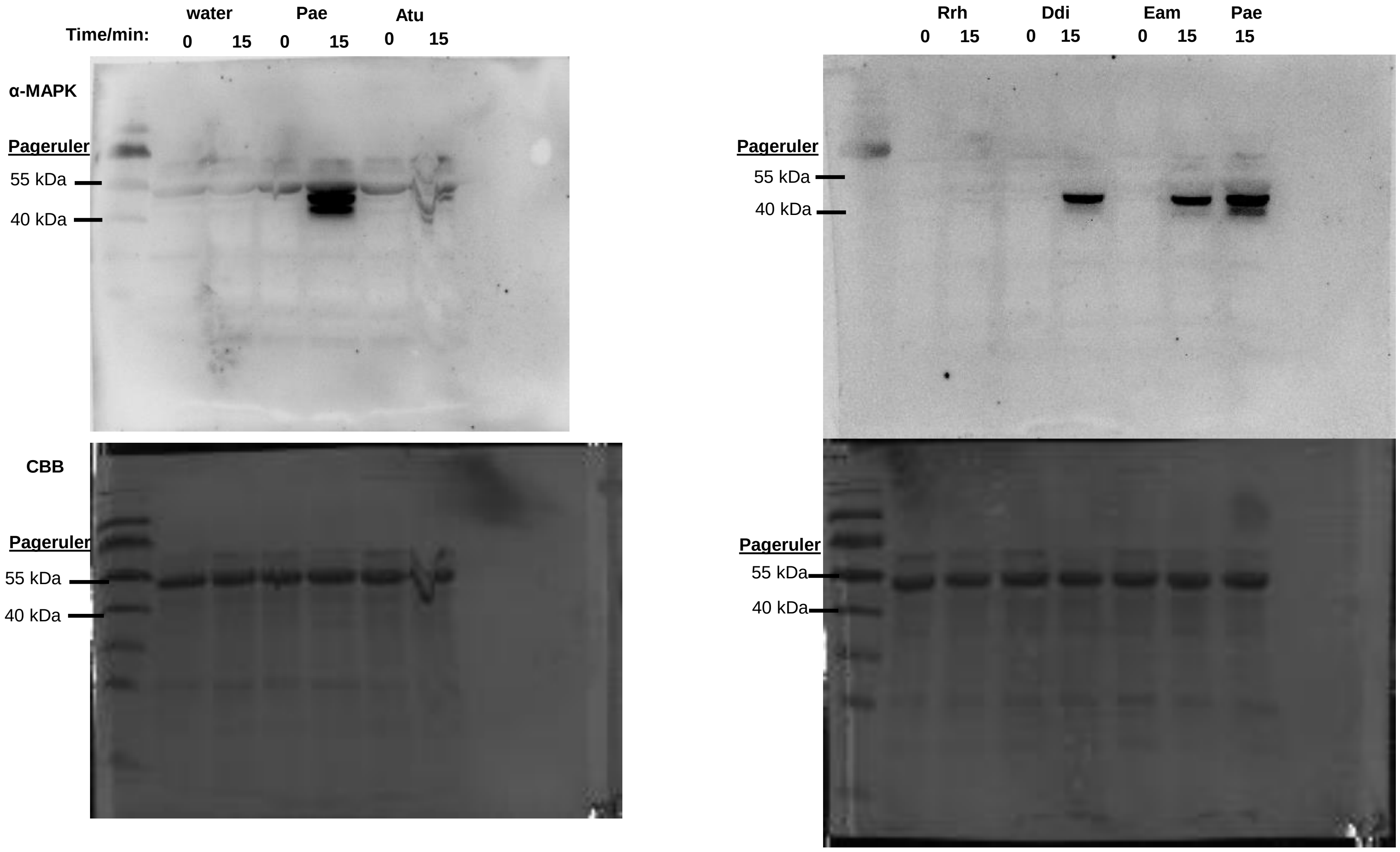

Figure 3

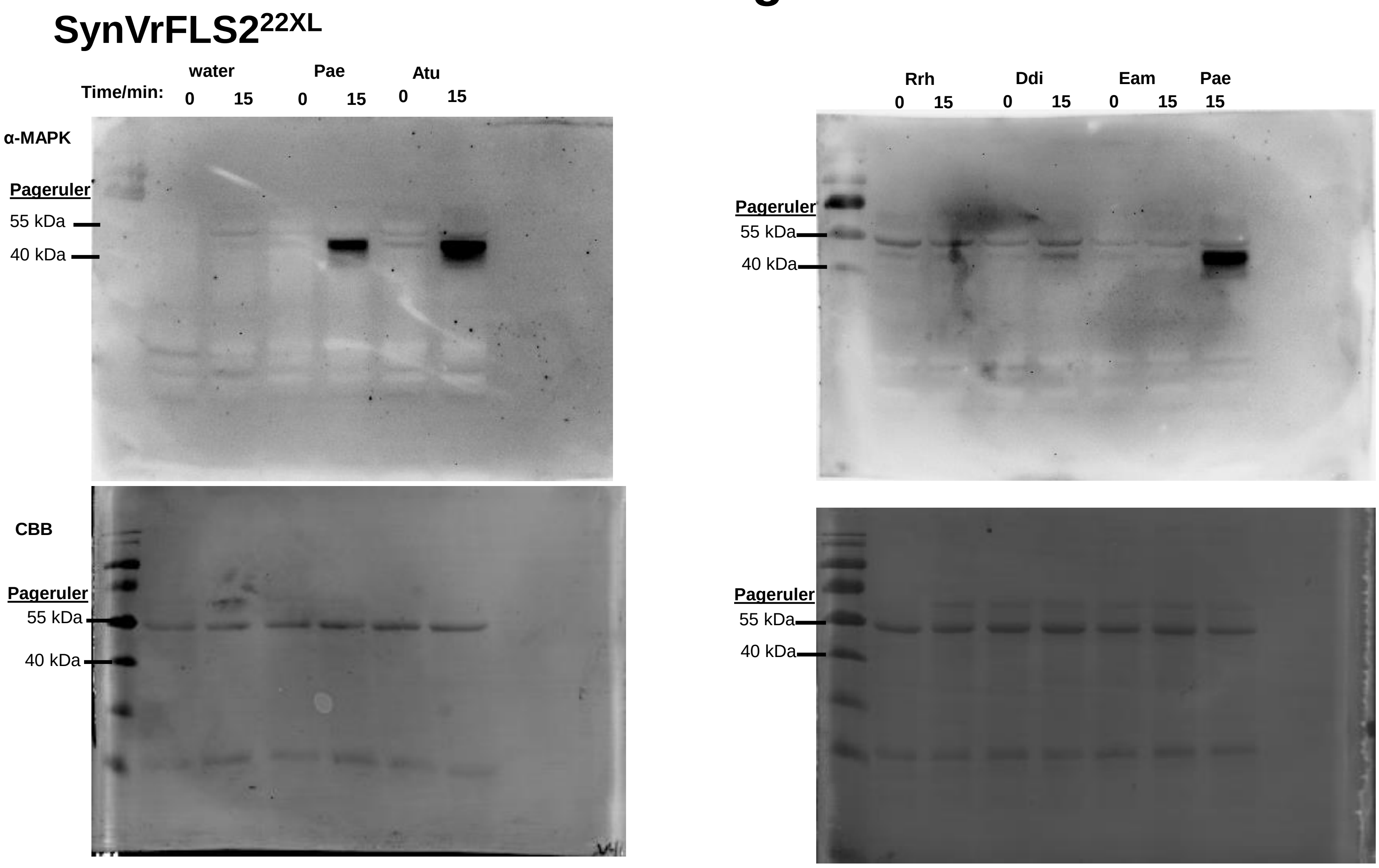

SynVrFLS2^{22XL}
water
Pae
Atu
Time/min:
0
15
0
15
0
15
α-MAPK
Pageruler
55 kDa
40 kDa
CBB
Pageruler
55 kDa
40 kDa
Rrh
Ddi
Eam
Pae
0
15
0
15
0
15
15
Pageruler
55 kDa
40 kDa
Pageruler
55 kDa
40 kDa

Extended Figure 2

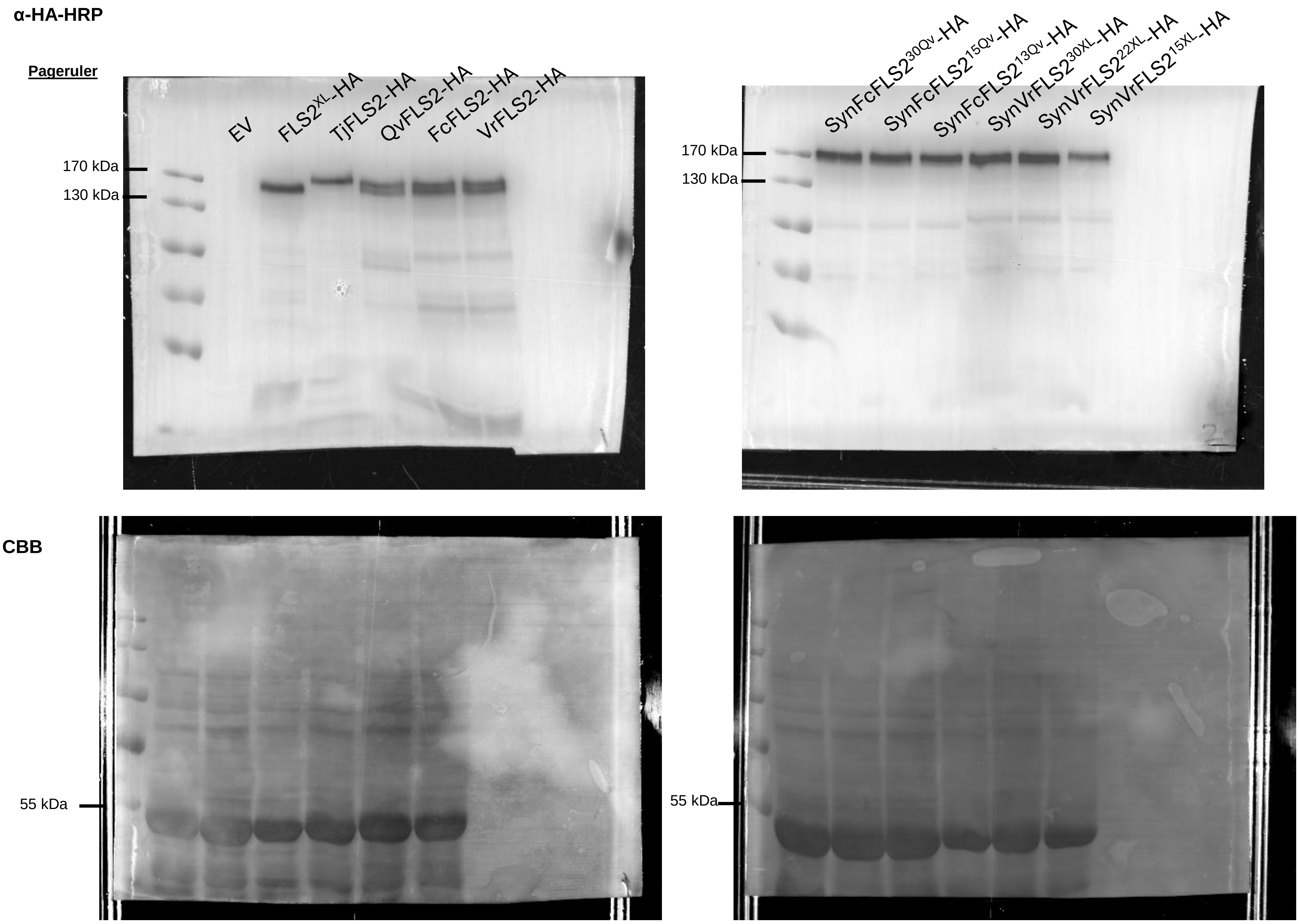

Extended Figure 3

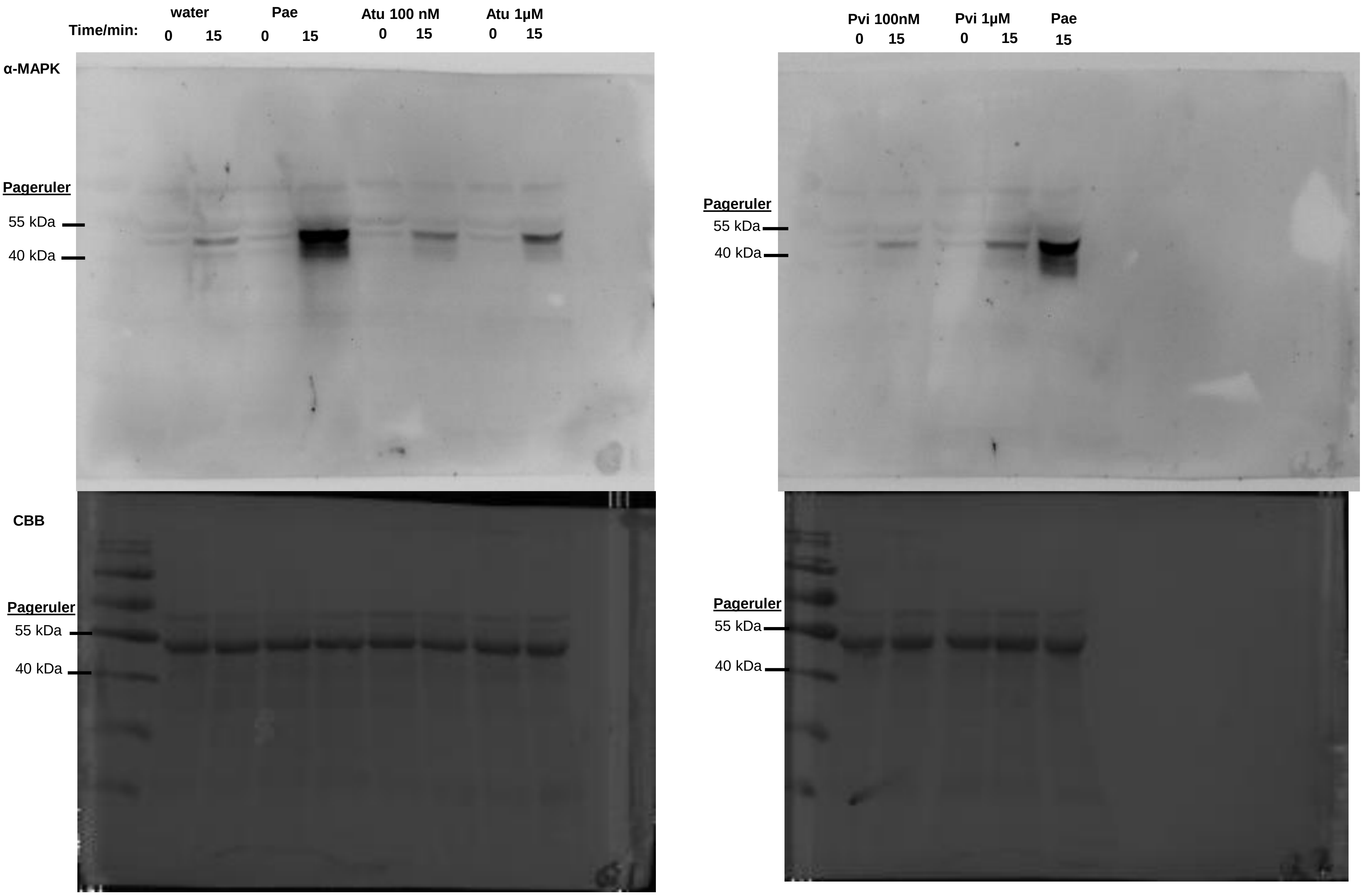

Supplement: Supplementary file 6 — Unprocessed and uncropped western blots. [file 41477_2025_2049_MOESM6_ESM.pdf]
